# Genetic modulation of oxycodone self-administration trajectories: from initiation to escalating burst patterns

**DOI:** 10.3389/fnbeh.2026.1913370

**Published:** 2026-08-28

**Authors:** Caleb I. Hodges, Eamonn P. Duffy, Jonathan O. Ward, Luanne H. Hale, Cove C. Andrews, Laura M. Saba, Marissa A. Ehringer, Ryan K. Bachtell

**Affiliations:** 1 Department of Psychology and Neuroscience, University of Colorado Boulder, Boulder, CO, United States; 2 Department of Integrative Physiology, University of Colorado Boulder, Boulder, CO, United States; 3 Institute for Behavioral Genetics, University of Colorado Boulder, Boulder, CO, United States; 4 Department of Pharmaceutical Sciences, Skaggs School of Pharmacy and Pharmaceutical Sciences, University of Colorado Anschutz Medical Campus, Aurora, CO, United States

**Keywords:** addiction, compulsive, drug loading, inbred, inter-infusion interval, substance abuse

## Abstract

**Introduction:**

Opioid Use Disorder (OUD) remains a prominent threat to global health. Genetic background influences the susceptibility of developing OUD, although specific genetic factors remain elusive. Rodent models that differ in susceptibility to escalation and dysregulation of opioid use are valuable tools to facilitate discovery of genetic pathways.

**Materials and methods:**

Phenotypes associated with the development of OUD were compared in seven classic inbred rat strains (M520/N, WKY/NCrl, F344/NCrl, F344/Stm, LEW/Crl, LEW/SSNHsd, LE/Stm) from the Hybrid Rat Diversity Panel (HRDP). A two-phase self-administration paradigm was utilized to assess characteristics of the acquisition of oxycodone self-administration during daily 2-h sessions, and the escalation of oxycodone use during daily 12-h sessions.

**Results:**

Genetic background influenced the acquisition of oxycodone self-administration as indicated by differences in the initiation of responding for oxycodone during each session and different amounts of oxycodone intake. We observed that escalation of oxycodone intake between-sessions was strain dependent, and the within-session distribution of oxycodone intake was strongly influenced by strain. The M520/N strain engaged in a unique pattern of intake, characterized by rapid initiation of oxycodone responding during the acquisition phase and a significant burst-like responding during escalation. Strain-dependent sex differences were also observed in several acquisition and escalation metrics. Of interest, rapid burst responding was more prevalent in females of the M520/N strain compared to males.

**Discussion:**

Together, these data indicate that genetic background influences not only overall oxycodone intake, but specific within- and between-session metrics that capture patterns of consumption across the substance use trajectory.

## 1 Introduction

Despite being one of the most prescribed drug classes in the United States, opioids are long recognized for their abuse potential (Fuentes et al., 2018). Prescription of opioids including oxycodone has been associated with the start of the opioid epidemic, a crisis that has resulted in approximately 6.7–7.6 million individuals in the United States developing opioid use disorder (OUD) (Keyes et al., 2022). In response to the opioid epidemic, opioid prescriptions have steadily declined since 2011 (Preuss et al., 2025). However, there is evidence suggesting that prescription opioids remain effective in certain populations where alternative treatments are lacking, such as in cancer patients (Harsanyi et al., 2023; Pergolizzi et al., 2021). Opioids are highly effective at producing acute analgesia, but long-term use of opioids can result in dependence, tolerance, and overdose (Ballantyne, 2015; Edlund et al., 2014; Gomes et al., 2021). Understanding which individuals in these populations are vulnerable or resistant to developing OUD will aid clinical decisions regarding pain treatment.

Although there is high abuse potential associated with opioids, OUD only occurs in a subset of individuals who regularly consume opioids (Degenhardt et al., 2019). Nearly 13% of people who used prescription opioids have misused opioids with approximately 16% of those individuals developing OUD (Substance Abuse and Mental Health Services Administration, 2017; Han et al., 2017). Heritability estimates of OUD range from 0.31 to 0.54, suggesting that individuals differ in their genetic susceptibility to developing problematic opioid use (Kendler et al., 1999; Kendler et al., 2000; Tsuang et al., 1998). Genome-wide association studies (GWAS) have identified few genetic variants associated with the development of OUD (Hancock et al., 2018). These datasets often rely on broad diagnostic criteria, which limits their ability to identify genetic variants associated with protection or vulnerability across different phases of drug use such as initiation, maintenance of drug use, or compulsive use that is observed in OUD (Miller et al., 2024). Animal models can provide insight into the genetic contribution by examining specific behavioral phenotypes relevant to the progression of OUD that cannot be readily assessed in human cross-sectional GWAS. For example, a recent GWAS conducted in heterogenous stock (HS) rats revealed that heroin consumption and extinction are heritable traits, and identified genetic variants associated with heroin consumption, escalation of intake, and motivation to obtain heroin (Kuhn et al., 2025).

Phenotypic characterization of opioid-related behaviors in genetically diverse rodent populations provides a powerful approach for identifying genetic factors that contribute to OUD. The Hybrid Rat Diversity Panel (HRDP) consists of nearly 100 genetically divergent inbred rat strains, including two recombinant inbred panels (HXB/BXH and FXLE/LEXF) as well as several classic inbred strains (Tabakoff et al., 2019). Genetic variation across HRDP strains makes it useful for investigating the genetic contributions to complex behavioral traits such as oxycodone-induced analgesia and self-administration (Doyle et al., 2023; Duffy et al., 2024a; Duffy et al., 2024b; Sharp et al., 2021; Sharp et al., 2024). Work in HS rats further demonstrates substantial individual variability in oxycodone self-administration, escalation, and addiction-like behaviors (Kallupi et al., 2026). Together, these rodent models provide complementary platforms for integrating behavioral phenotyping with genomic analyses to identify biological mechanisms contributing to OUD risk.

Intravenous drug self-administration (IVSA) assesses volitional drug intake in rodents with procedural variations such as short-access (ShA) and long-access (LgA) conditions modeling different phases of substance use disorders (Kuhn et al., 2019; Slosky et al., 2022). For example, ShA conditions often facilitate initiation of drug use or acquisition, and often display stabilized intake while LgA conditions promote escalation and compulsive use (Edwards and Koob, 2013; Kuhn et al., 2019). Within-session patterns of drug self-administration have become increasingly recognized as an important factor in characterizing drug intake throughout the different phases. Belin et al. (2009) found that within-session distribution of cocaine infusions was positively correlated with addiction severity. Moreover, the emergence of a burst-like pattern of responding, where several infusions are delivered within a short duration, predicted high cocaine use severity (Belin et al., 2009). Recent studies have observed burst-like patterns of heroin self-administration under specific self-administration parameters where rats have periods of free access to heroin (D’Ottavio et al., 2023; D’Ottavio et al., 2025a). Analysis of other within-session parameters, such as inter-infusion intervals, suggests that short bursts of oxycodone intake correlate with the reinstatement of oxycodone seeking (Guha et al., 2022). Little is known about the genetic factors contributing to within-session regulation of drug intake that may reflect the emergence of dysregulated and uncontrolled oxycodone use.

In this study, we utilized a two-phase IVSA paradigm to characterize oxycodone use in male and female rats from several “classic” inbred strains that we previously characterized (Duffy et al., 2024a). The inbred rat strains being assessed include a subset of classic, non-recombinant inbred strains that displayed robust escalation of oxycodone intake under LgA conditions (Duffy et al., 2024a). Here, we further describe within-session phenotypes associated with the acquisition (ShA) and escalation (LgA) phases of the paradigm. Acquisition was assessed using between-sessions measures such as infusions per session and lever discrimination while within-session measures include early session metrics that measure the initiation of oxycodone responding during each ShA session. Escalation was assessed using the between-session measures of the number of infusions per session and the magnitude and rate of escalation, as well as within-session measures such as inter-infusion intervals and burst responding. Combining these between- and within-session metrics across both phases enables us to identify genetic differences between specific phenotypes across the trajectory of oxycodone use. We hypothesized that we would identify differences between the strains in some of the within-session metrics that were not captured by the between-session metrics. While all strains acquired and escalated oxycodone intake, only the M520/N strain displayed a rapid transition to uncontrolled oxycodone use marked by higher burst responding compared to the other strains. These findings suggest that there are genetic influences that contribute to different phases of opioid use and specific characteristics of opioid use patterns.

## 2 Materials and methods

### 2.1 Subjects

Adult (PND60+) male and female rats (*n* = 193) from seven inbred strains were tested in this experiment (Table 1). The WKY/NCrl, F344/NCrl, and LEW/Crl strains were obtained from Charles River. The LEW/SSNHsd (LEW/SS) strain was obtained from Envigo/Inotiv. The remaining strains (M520/N, F344/Stm, LE/Stm) were obtained from the Medical College of Wisconsin (provided by Dr. Melinda Dwinell, R24OD024617). Rats were allowed to habituate for at least 1 week following arrival before beginning any procedures. Prior to surgery and the self-administration paradigm, animals were tested for somatosensory and analgesia-related measures (Duffy et al., 2024b). Rats were single-housed in a temperature (22°C) and humidity (40%) controlled vivarium that had a 12-h light and 12-h dark cycle. Standard rat chow (Teklad 2918, Envigo/Inotiv, Indianapolis, IN) and water were provided *ad libitum*. Animals were tested in 12 cohorts (*n* = 4–24/cohort). All procedures were approved and conducted in accordance with institutional guidelines outlined by the Institutional Animal Care and Use Committee at the University of Colorado Boulder, an Association for Assessment and Accreditation of Laboratory Animal Care International accredited institution.

**TABLE 1:** Sample size per strain and sex.

Strain	RRID	Oxycodone		Saline	
		M	F	M	F
M520/N	RRID:RRRC_00168	8	9	6	7
WKY/NCrl	RRID:RGD_1358112	7	7	8	8
F344/NCrl	RRID:RGD_737926	9	8	8	8
F344/Stm	RRID:RGD_1302686	10	5	10	6
LEW/SSNHsd	RRID:RGD_737922	6	6	5	4
LEW/Crl	RRID:RGD_737932	5	5	6	5
LE/Stm	RRID:RGD_629485	4	8	7	7
Total		49	48	50	45

### 2.2 Intravenous catheter surgeries

**FIGURE 1:**
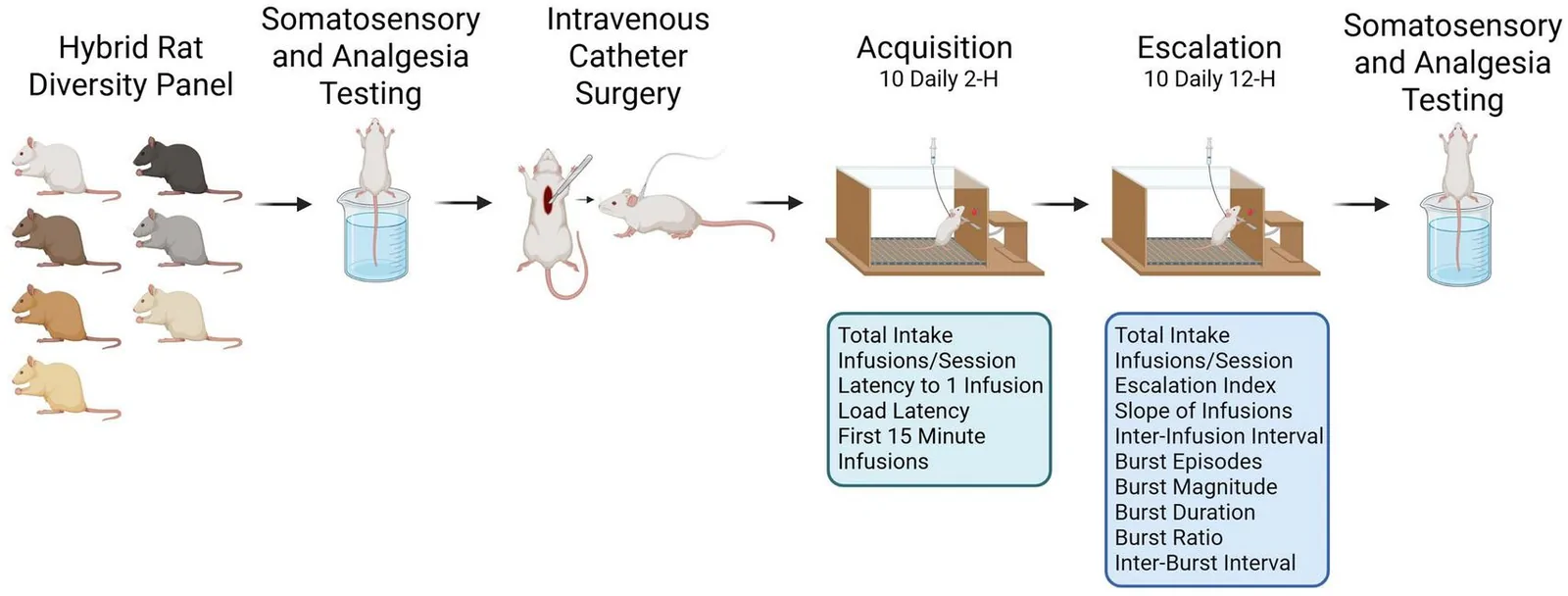
Overview of longitudinal behavioral protocol. Schematic depicting the sequencing of the behavioral phenotyping and surgical procedures. Rats underwent somatosensory and analgesia testing as previously reported before surgical implantation of intravenous catheters (Duffy et al., 2024b). Oxycodone or saline intake was evaluated using a self-administration procedure with a fixed-ratio 1 schedule of reinforcement. During the acquisition phase, rats lever pressed for saline/oxycodone in ten daily 2-h (ShA) sessions and behavioral phenotypes were included to evaluate changes in reinforcement learning. The escalation phase was comprised of 10 daily 12-h (LgA) sessions and monitored between session changes in intake as well as within-session metrics to assess the temporal organization and dysregulation of drug intake. Rats were subsequently tested in the same somatosensory and analgesia tests (data not shown). Created in BioRender. Hodges (2026): https://BioRender.com/ew7b43v.

Jugular indwelling catheters were implanted utilizing previously described procedures (O’Neill et al., 2012). Briefly, isoflurane (2–4%) was used to anesthetize animals. Intravenous catheters were inserted into the right jugular vein under aseptic conditions. After isolation of the vein, a 22-gaige needle was used to puncture the vein and allow for catheter tubing implantation which was secured using suture threads. Sterile catheters (Access Technologies, Skokie, IL) consisted of 14 cm of polyurethane tubing (0.51 mm inner diameter, 1.12 mm outer diameter) secured to a 22-gauge back mount pedestal (Protech International). The analgesic carprofen (5 mg/kg, s.c.) and antibiotic enrofloxacin (5 mg/kg, s.c.) were administered immediately prior to surgery. Daily subcutaneous injections of carprofen (5 mg/kg) were administered for 2 days following surgery while monitoring animal health. Catheters were flushed daily with 0.2 mL heparinized (20 IU/mL) bacteriostatic saline containing gentamicin sulfate (0.33 mg/mL; Hospira) until self-administration procedures began. Catheter patency was ensured using 0.2 mL propofol (10 mg/mL propofol, Sagent) solution administered before the acquisition phase, prior to the escalation phase, and following the escalation phase to ensure patency of the catheters. Prior to self-administration, rats were assigned to the saline or oxycodone group using a pseudorandomized procedure based on analgesic testing (Figure 1; Sharp et al., 2021).

### 2.3 Oxycodone self-administration

Self-administration sessions were conducted in operant chambers (Med Associates, St. Albans, VT) equipped with two retractable levers, a stimulus cue light above each lever, and a sound-attenuating fan. Self-administration sessions began at the start of the dark phase of the dark/light cycle. The acquisition and escalation of oxycodone (Oxycodone HCl; B&B pharmaceutical, Englewood, CO) use was measured in a two-phased self-administration paradigm. The acquisition phase utilized a fixed-ratio 1 (FR1) reinforcement schedule in ten daily 2-h ShA sessions. A response at the active lever resulted in either an oxycodone infusion (0.15 mg/kg/infusion) or saline infusion (0.9%, 0.15 mL/infusion) delivered over 5-s and illumination of a cue light (7.5-W white light, 20-s). A 20-s timeout period (TO20) followed each infusion. Inactive lever responses were recorded but did not result in an infusion or cue light activation. The escalation phase consisted of a similar reinforcement schedule (FR1:TO20) to the acquisition phase. The escalation phase was conducted during 10 daily 12-h LgA sessions with a 2-d break between sessions 5 and 6.

### 2.4 Behavioral phenotypes

#### 2.4.1 Number of infusions and total intake

The number of infusions within each session was used to evaluate the overall pattern of intake during the acquisition and escalation phases. We used the number of infusions delivered within the first 15-m of each 2-h session to measure the initiation of oxycodone taking during each ShA session. The total number of infusions was also used to calculate total oxycodone intake (mg/kg) by multiplying the number of infusions by the unit dose (0.15 mg/kg/infusion) during each phase. These measures provided an overview of intake trends between the strains as previously reported (Duffy et al., 2024a).

#### 2.4.2 Latency to the first infusion

Latency to the first infusion was determined by recording the time the rat took to press the active lever following the onset of the session. This measure was used to characterize the initiation of each oxycodone self-administration session during the acquisition phase. The latency to initiate each session is anticipated to decrease as rodents learn the goal-directed behavior (Rode et al., 2020). Progressive decreases in latencies across sessions reflects the consolidation of the goal-directed behavior between sessions rather than within-session learning.

#### 2.4.3 Load time

Drug self-administration sessions are often initiated with a drug loading phase (e.g., front-loading) in which rats engage in a high rate of responding at the beginning of self-administration sessions (Ahmed and Koob, 1998; Ahmed et al., 2000). The loading phase is an index of drug reinforcement and the regulation of the rat’s preferred drug concentrations (D’Ottavio et al., 2025a; Guha et al., 2022). To quantify the drug loading phase, we calculated “Load Time” by subtracting the latency to the first infusion from the latency to deliver three infusions. This metric was utilized to characterize early session responding during the acquisition phase as a measure of drug regulation coinciding with learning.

#### 2.4.4 Escalation metrics

An escalation index was utilized to determine the magnitude of escalation of oxycodone infusions throughout the escalation phase (Kallupi et al., 2026). The escalation index was calculated by averaging the total number of infusions during the last three LgA sessions and dividing by the average number of infusions from the first three sessions of LgA. Normalizing later intake to early intake controls for baseline differences between strains and allows for direct strain comparisons in the escalation of intake.


$$\frac{Avg.Infusions:LastThreeSessions}{Avg.Infusions:FirstThreeSessions}=E\,s\,c\,a\,l\,a\,t\,i\,o\,n\,I\,n\,d\,e\,x$$

Regression-derived slopes of infusions across the escalation phase were used to complement the escalation index measure. The slope of infusions captures the rate of escalation and incorporates infusion data from all sessions rather than select “early” and “late” sessions but assumes that there is a linear relationship between session and number of infusions. A slope of 0 indicates stable intake while a positive slope indicates progressive escalation and a negative slope indicates reduced intake over time.

#### 2.4.5 Inter-infusion interval

The inter-infusion interval was used as a measure of temporal organization of drug taking during the escalation phase (Panlilio et al., 2003). The average inter-infusion interval was calculated as the average time elapsed between successive infusions during the escalation phase. Progressive shortening of inter-infusion intervals across the escalation phase indicates that escalation of drug use coincides with within-session adaptations resulting from motivation, tolerance, and/or behavioral control (Belin et al., 2009; Garcia et al., 2020; Guha et al., 2022).

#### 2.4.6 Burst responding

Previous reports have indicated that rats engage in dysregulated drug intake by self-administering short bursts of infusions followed by a long drug-free interval (Allain et al., 2015; Allain et al., 2017; Belin et al., 2009; Brown et al., 2022). To capture “burst responding,” raw output data files containing the cumulative records of each timestamped behavioral event (e.g., active/inactive response, infusion, time-out responses) were used. The raw data from these files were extracted, and a custom MATLAB (Mathworks, R2025a, 25.1.0) script was used to determine the number of burst episodes, the average infusions per burst episode, inter-burst intervals, and the proportion of total infusions that occurred during a burst episode (Bachtell, 2026). Burst episodes were identified using two complementary temporal definitions to distinguish rapid, high-intensity bouts of intake from more prolonged episodes of self-administration. For the rapid burst analysis, a burst episode was defined as a minimum of three infusions occurring within a 90-s period. The sustained burst analysis defined a burst episode as a minimum of three infusions occurring within a 5-min period consistent with previous reports (Belin et al., 2009; D’Ottavio et al., 2025a). Under both definitions, once the minimum criterion was met, the burst episode continued until the rat no longer satisfied the temporal criterion, resulting in burst episodes that varied in size and duration. Burst magnitude was quantified as the number of infusions per burst episode, and burst duration was calculated as the elapsed time between the first and last infusion within each burst. We also determined the time elapsed between consecutive burst episodes (inter-burst interval), analogous to inter-infusion intervals. Lastly, to determine how strains differentially distribute their infusions in burst episodes vs. single discrete events, we calculated the ratio of infusions within burst episodes to total infusions by dividing the number burst infusions by the total number of infusions for each rat. A higher ratio of burst responding would indicate that more infusions were self-administered as part of a burst episode.

#### 2.4.7 Non-specific behavioral responding

To identify potential strain differences in non-specific motoric activity, we evaluated inactive lever responses and responses occurring during the 20-s timeout period. Inactive lever responding provides an index of non-specific behavioral regulation and discriminative control over operant responding. Timeout responding provides a measure of perseverative responding when oxycodone is unavailable following an infusion. To account for differences in the number of oxycodone infusions and corresponding timeout periods, the number of timeout responses was normalized to the number of drug infusions using the following equation:


$$\frac{T\,i\,m\,e\,o\,u\,t\,R\,e\,s\,p\,o\,n\,s\,e\,s}{I\,n\,f\,u\,s\,i\,o\,n\,s}=N\,o\,r\,m\,a\,l\,i\,z\,e\,d\,T\,i\,m\,e\,o\,u\,t\,R\,e\,s\,p\,o\,n\,d\,i\,n\,g$$

### 2.5 Data analysis

All data are presented as the mean ± standard error of the mean (SEM). Data were analyzed and graphed using both R (v.4.5.3) and GraphPad Prism (v.11.0.1). Prior to analysis, animals were excluded based on a set of predefined criteria. Rats were removed from the dataset if they experienced health complications (e.g., > 20% weight loss, infection, etc.), catheter failure, missing more than 3 sessions in either the acquisition or escalation phase, or failure to complete LgA session 10 of the escalation phase. If data from a single session was missing (e.g., computer failure, tether failure, animal health, etc.), that data point was imputed by calculating the mean of the immediately preceding and subsequent session. Summary measure data points that were > 2 standard deviations (SD) from the strain mean were considered outliers and winsorized as the strain mean ± 2 SD. One M520/N rat was excluded from all analyses because it failed to acquire the self-administration behavior (zero oxycodone infusions) during the acquisition phase.

Longitudinal measures collected across sessions (e.g., infusions, latency to 1st infusion, burst episodes, etc.) were initially analyzed using comprehensive linear mixed-effects models. These included a global 4-way linear mixed-effects model with Strain, Group, and Sex as categorical factors, and Session as a continuous variable, and a reduced 3-way model excluding sex. Although these models provided an overall assessment of experimental effects, interpretation was limited by multiple higher-order interactions, especially involving the Group factor. Given this complexity, subsequent analyses focused on simplified models to better resolve biologically meaningful effects. Thus, separate 2-way linear mixed-effects models were conducted within each self-administering group to examine the effect of Strain across Sessions, and the interactive effects of Strain and Session. Separate 2-way linear mixed-effects models were also conducted within each strain to examine the effect of Sex across sessions. And the interactive effects of Sex and Session. The Geisser–Greenhouse correction was used to correct for any potential deviations from sphericity. Significant interactions were followed by *post hoc* comparisons using a Tukey’s multiple comparison test to control for familywise error. Summary measures (e.g., total oxycodone intake, escalation index, average inter-infusion interval, regression-derived slopes, etc.) were also used to analyze strain differences using separate one-way ANOVAs within each Group (Saline or Oxycodone) followed by pairwise strain comparisons. To evaluate the relationship between the acquisition, escalation, and dysregulated oxycodone use phenotypes, we computed Pearson correlation coefficients for several summary measures. Correlations were examined at the level of individual rats regardless of strain.

To further quantify changes in acquisition and escalation metrics (e.g., infusions, latency to 1st infusion, load time, inter-infusion intervals, etc.), linear regression analyses were conducted for each subject with the slope of the regression line serving as an index of change. An F-test was used to determine whether the slope significantly differed from zero, and to compare slopes between Groups or Sex within each Strain. The escalation index was also analyzed using a one-sample *t*-test to determine if these measures differed from a theoretical value of 1 (no escalation). For all tests, statistical significance was set at *p* < 0.05.

## 3 Results

### 3.1 Strain differences in the acquisition of oxycodone and saline self-administration

**FIGURE 2:**
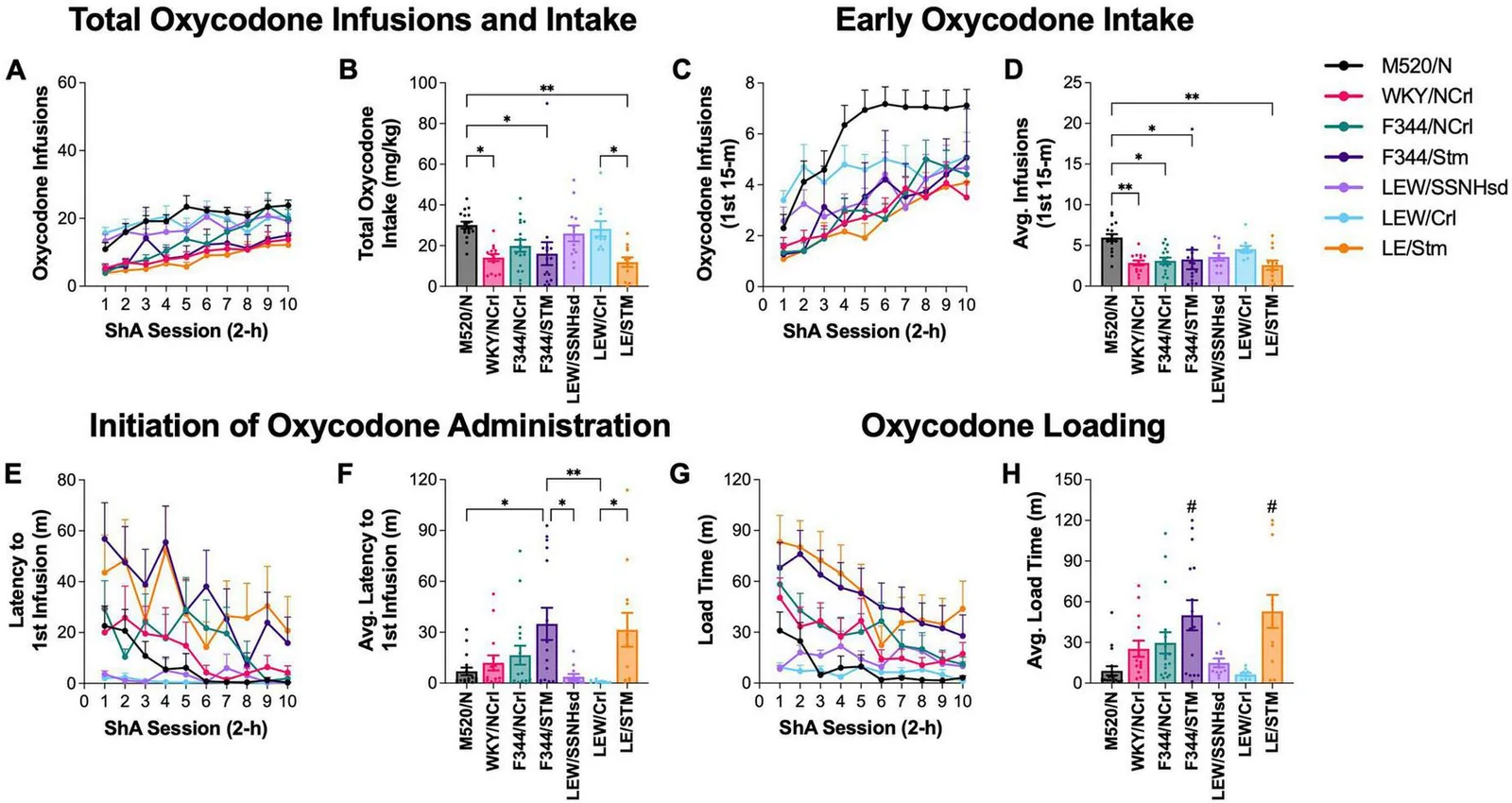
Strain differences in the acquisition and early-session regulation of oxycodone self-administration. **(A)** Mean ( ± SEM) number of oxycodone infusions per 2-h (Short-access; ShA) session across the 10 daily ShA acquisition sessions for all seven inbred rat strains. Mixed-effects analyses revealed significant main effect of Strain (*F*_6,__90_ = 4.47, *p* < 0.001) and Session (*F*_1.792,__161.2_ = 18.97, *p* < 0.001). **(B)** Total oxycodone intake (mg/kg) throughout the acquisition phase also revealed significant strain differences (*p < 0.05; **p < 0.01). **(C)** Mean ( ± SEM) number of oxycodone infusions delivered during the first 15 min of each 2-h ShA session. Mixed-effects analyses revealed significant main effect of Strain (*F*_6,__90_ = 3.89, *p* < 0.002) and Session (*F*_1.915,__172.4_ = 21.35, *p* < 0.001). **(D)** Significant strain differences were also observed on the average number of infusions during the first 15 min across all sessions (*p < 0.05; **p < 0.01). **(E)** The initiation of oxycodone self-administration was measured by the latency (m) to the 1st oxycodone infusion across the 10 ShA sessions. Mixed-effects analyses revealed a significant main effect of Strain (*F*_6,__90_ = 4.58, *p* < 0.001) and Session (*F*_2.912,__264.4_ = 6.18, *p* < 0.001). **(F)** The average latency (minutes) to the 1st infusion across the acquisition phase also significantly varied between strains (*p < 0.05; **p < 0.01). **(G)** Analysis of the oxycodone load time (m) across the 10 ShA sessions further indicated significant main effect of Strain (*F*_6,__90_ = 5.65, *p* < 0.001) and Session (*F*_1.987178.8_ = 14.86, *p* < 0.001). **(H)** Strains differed in the average load time (minutes) across the acquisition phase with the F344/Stm and LE/Stm showing significantly longer load times compared with M520/N, LEW/SS, and LEW/Crl (# *p* < 0.05). *N* = 10–17/strain.

Rats from seven inbred strains were trained to self-administer oxycodone or saline during 10 daily 2-h short-access (ShA) sessions (Figure 1). Acquisition of oxycodone self-administration differed markedly across strains (Figure 2A). Mixed-effects analyses on the number of oxycodone infusions during the acquisition phase revealed significant main effect of Strain (*F*_6,__90_ = 4.47, *p* < 0.001) and Session (*F*_1.792,__161.2_ = 18.97, *p* < 0.001). Tukey *post-hoc* comparisons revealed that M520/N strain had significantly higher oxycodone intake compared to the WKY/NCrl, F344/Stm, and LE/Stm strains, while oxycodone intake in the LEW/Crl strain was higher than the LE/Stm strain (Figure 2B; *p* < 0.05). Linear regression analyses confirmed these findings as indicated by significant positive slopes in the M520/N, WKY/NCrl, F344/NCrl, and LE/Stm strains, while the F344/Stm, LEW/Crl, and LEW/SS strains showed more subtle positive slopes that did not reach statistical significance (Table 2).

**TABLE 2:** Linear regression analyses for longitudinal acquisition metrics in oxycodone self-administering rats.

Measure	Strain	Linear regression results
Acquisition: infusions	M520/N	*b* = 1.11, *p* < 0.001*
	WKY/NCrl	*b* = 0.92, *p* < 0.001*
	F344/NCrl	*b* = 1.99, *p* < 0.001*
	F344/Stm	*b* = 0.89, *p* = 0.063
	LEW/SS	*b* = 0.69, *p* = 0.052
	LEW/Crl	*b* = 0.31, *p* = 0.351
	LE/Stm	*b* = 1.01, *p* < 0.001*
Acquisition: first 15 min	M520/N	*b* = 0.47, *p* < 0.001*
	WKY/NCrl	*b* = 0.27, *p* < 0.001*
	F344/NCrl	*b* = 0.41, *p* < 0.001*
	F344/Stm	*b* = 0.38, *p* = 0.011*
	LEW/SS	*b* = 0.22, *p* = 0.005*
	LEW/Crl	*b* = 0.10, *p* = 0.181
	LE/Stm	*b* = 0.34, *p* < 0.001*
Acquisition: latency to 1st infusion	M520/N	***b*** = −2.48, *p* < 0.001*
	WKY/NCrl	***b*** = −2.52, *p* = 0.004*
	F344/NCrl	***b*** = −2.35, *p* = 0.017*
	F344/Stm	***b*** = −4.69, *p* = 0.007*
	LEW/SS	*b* = −0.85, *p* = 0.051
	LEW/Crl	***b*** = −0.20, *p* = 0.004*
	LE/Stm	*b* = −2.53, *p* = 0.101
Acquisition: load time	M520/N	***b*** = −2.74, *p* < 0.001*
	WKY/NCrl	***b*** = −3.84, *p* < 0.001*
	F344/NCrl	***b*** = −4.30, *p* < 0.001*
	F344/Stm	***b*** = −5.22, *p* < 0.001*
	LEW/SS	*b* = −0.15, *p* = 0.814
	LEW/Crl	*b* = −0.49, *p* = 0.117
	LE/Stm	***b*** = −5.86, *p* < 0.001*

Significance indicated by emboldened text and asterisk (*p < 0.05).

To determine whether strains differed in their initial regulation of oxycodone intake during the acquisition phase, we quantified the number of infusions during the first 15-m of each session, latency to the first infusion, and load time (Figures 2C–H). Mixed-effects analyses on the number of oxycodone infusions during the first 15-m of each session revealed significant main effect of Strain (*F*_6,__90_ = 3.89, *p* < 0.002) and Session (*F*_1.915,__172.4_ = 21.35, *p* < 0.001). Tukey *post-hoc* comparisons revealed that M520/N strain had significantly higher early session oxycodone intake compared with all strains except LEW/SS and LEW/Crl (Figure 2D; *p* < 0.05). Linear regression modeling indicated that all strains except the LEW/SS strain significantly increased the number of oxycodone infusions administered in the first 15-m of each ShA session (Table 2).

The initiation of oxycodone self-administration in each session was assessed using the latency to the 1st infusion (Figures 2E,F). Linear regression analyses support a progressive shortening in the latency to the first infusion in all strains except the LEW/SS and LE/Stm strains (Table 2). Mixed-effects analyses on the latency to the 1st infusion also revealed significant main effect of Strain (*F*_6,__90_ = 4.58, *p* < 0.001) and Session (*F*_2.912,__264.4_ = 6.18, *p* < 0.001). Tukey *post-hoc* comparisons revealed that the F344/Stm had significantly longer latencies to initiate oxycodone self-administration compared with the M520/N, LEW/SS, and LEW/Crl strains, while the LE/Stm strain was significantly longer compared with the LEW/Crl strain (Figure 2F; *p* < 0.05).

We next characterized how rats regulated oxycodone intake at the beginning of the session by calculating the drug loading time (Figures 2G,H). Drug loading is a phenomenon observed in IVSA studies in which rats engage in a high rate of responding at the beginning of self-administration sessions to rapidly reach desired drug levels (Ahmed and Koob, 1998; Ahmed et al., 2000). Linear regression modeling indicated that all, but the LEW/Crl and LEW/SS, strains showed significant shortening of oxycodone loading times throughout the acquisition phase (Table 2). Mixed-effects analyses on oxycodone load times revealed significant main effect of Strain (*F*_6,__90_ = 5.65, *p* < 0.001) and Session (*F*_1.987178.8_ = 14.86, *p* < 0.001). Tukey *post-hoc* comparisons revealed that both the F344/Stm and LE/Stm had significantly longer load times compared with the M520/N, LEW/SS, and LEW/Crl strains (Figure 2H; *p* < 0.05).

Similar acquisition metrics were analyzed for saline self-administering rats (Supplementary Figure 1 and Supplementary Table 1). The only acquisition metric that displayed statistically significant differences in the linear mixed model was the latency to 1st infusion where we detected a significant main effect of Strain (*F*_6,__88_ = 2.99, *p* = 0.02) and Session (*F*_2.847,__250.5_ = 4.57, *p* = 0.005). Tukey *post-hoc* comparisons revealed that the LE/Stm strain had significantly longer latencies to initiate saline self-administration compared with all other strains, and the M520/N strain displayed longer latencies that the F344/N and LEW/Crl strains (Supplementary Figure 1F; *p* < 0.05).

Together, these findings demonstrate that genetic background influences not only the amount of oxycodone consumed during acquisition phase but also the initiation and regulation drug intake at the beginning of each session. Notably, M520/N, LEW/SS, and LEW/Crl strains displayed the most robust acquisition phenotypes, characterized by the higher total intake, high early session intake, and rapid initiation and loading of oxycodone self-administration.

### 3.2 Strain differences in the escalation of oxycodone and saline self-administration

**FIGURE 3:**
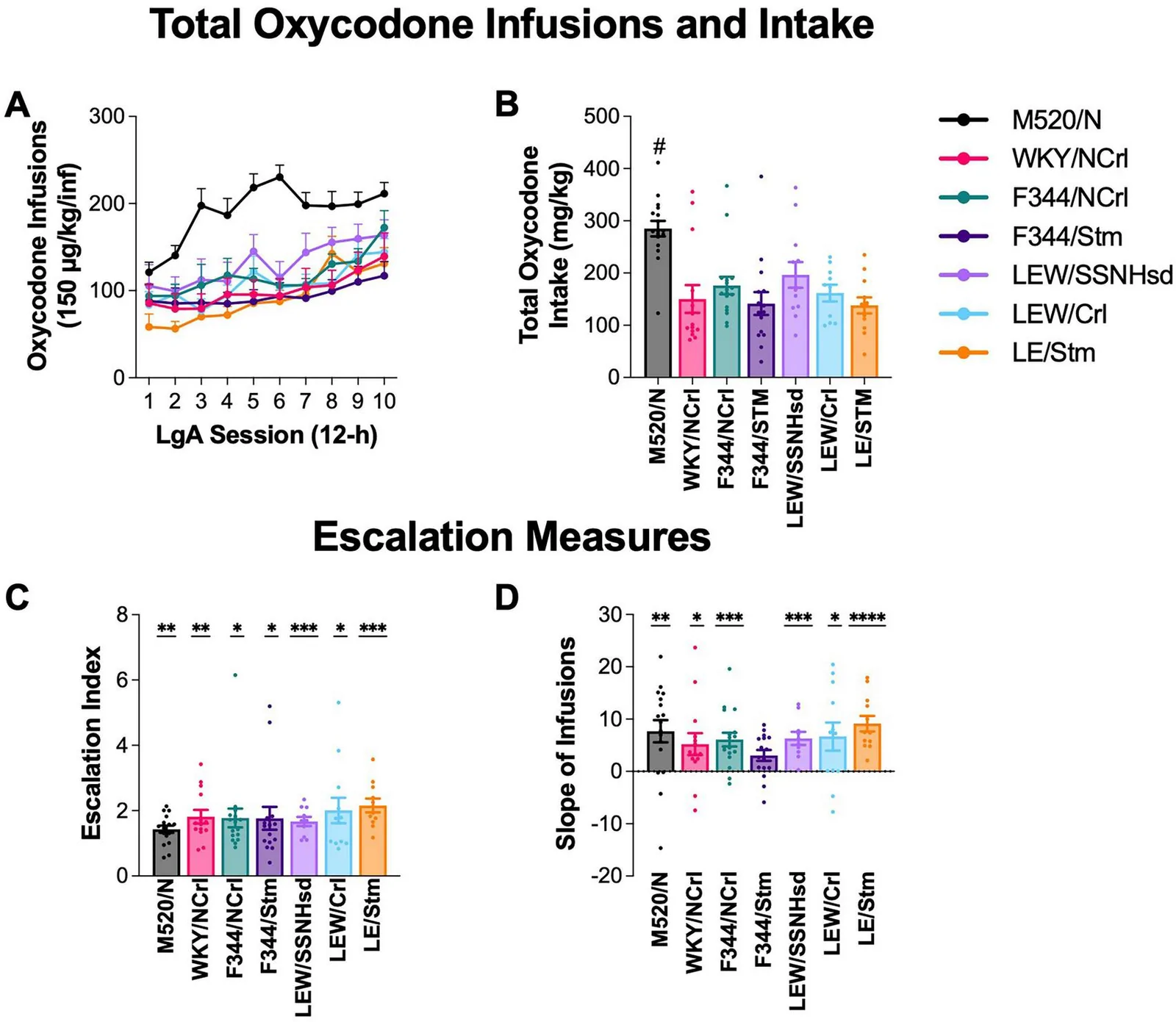
Strain differences in the escalation of oxycodone self-administration. **(A)** Mean ( *SEM*) number of oxycodone infusions per 12-h (Long-access; LgA) session across the 10 daily 12-h LgA escalation sessions for seven inbred rat strains. Oxycodone intake varied between the strains across the escalation phase as indicated by a significant Strain × Session interaction (*F*_14.4,__201.2_ = 1.92, *p* = 0.025). The M520/N strain displayed greater oxycodone intake earlier in the escalation phase compared to other strains. **(B)** Total oxycodone intake (mg/kg) throughout the escalation phase also revealed significant strain differences with the M520/N strain displayed significantly higher total intake compared to all other strains (#*p* < 0.05). **(C)** An escalation index was calculated for each strain and was compared to a hypothetical value of 1.0 (i.e., no change indicated by dotted line) using a one-sample *t*-test. The average escalation index for all strains showed significant escalation (*p < 0.05; **p < 0.01; ***p < 0.001). **(D)** Linear regression slopes representing the rate of increase in oxycodone infusions over sessions were also evaluated for significant non-zero positive slopes (intake escalation). All strains except the F344/Stm displayed significant intake escalation rates (*p < 0.05; **p < 0.01; ***p < 0.001; ****p < 0.0001). *N* = 10–17/strain.

Following acquisition, rats underwent ten daily 12-h long-access (LgA) self-administration sessions to promote escalation of oxycodone intake. Linear regression and mixed-effects analyses demonstrated robust escalation of oxycodone intake in all strains (Table 3 and Figure 3). A significant Strain × Session interaction was observed (*F*_14.4,__201.2_ = 1.92, *p* = 0.025) suggesting that oxycodone intake varied between the strains across the escalation phase. *Post hoc* comparisons indicate that the M520/N strain displayed greater oxycodone intake earlier in the escalation phase compared to other strains (Figure 3A). Analysis of total oxycodone intake during the escalation phase revealed a significant difference between strains (Figure 3B; one-way ANOVA, *F*_6,__90_ = 7.53, *p* < 0.001) with the M520/N strain consuming significantly more oxycodone than all other strains. To further evaluate whether strains differed in the magnitude or rate of escalation independent of baseline intake, we compared two complementary measures: the escalation index and the linear regression slope of oxycodone intake (Figures 3C,D). Despite large strain differences in total oxycodone consumption, neither the escalation index (*F*_6,__89_ = 0.761, *p* = 0.603) nor escalation slope (*F*_6,__91_ = 0.978, *p* = 0.445) differed among strains. Intake escalation was confirmed using a one-sample *t*-test to determine if the average escalation index for each strain was significantly different than a hypothetical magnitude of one (i.e., no change/escalation). All strains displayed significant intake escalation (Figure 3C). Linear regression modeling further confirmed significant intake escalation with positive slopes for all strains except the F344/Stm strain (Figure 3D and Table 3).

**TABLE 3:** Linear regression analyses for longitudinal escalation metrics in oxycodone self-administering rats.

Measure	Strain	Linear regression results
Escalation: infusions	M520/N	*b* = 7.69, *p* < 0.001*
	WKY/NCrl	*b* = 5.78, *p* = 0.008*
	F344/NCrl	*b* = 6.45, *p* < 0.001*
	F344/Stm	*b* = 3.19, *p* = 0.066
	LEW/SS	*b* = 7.44, *p* < 0.001*
	LEW/Crl	*b* = 6.30, *p* < 0.001*
	LE/Stm	*b* = 9.35, *p* < 0.001*
Escalation: inter-infusion intervals	M520/N	***b*** = −0.27, *p* < 0.001*
	WKY/NCrl	***b*** = −0.79, *p* < 0.001*
	F344/NCrl	***b*** = −0.57, *p* < 0.001*
	F344/Stm	*b* = −0.07, *p* = 0.937
	LEW/SS	***b*** = −0.66, *p* < 0.001*
	LEW/Crl	***b*** = −0.61, *p* < 0.001*
	LE/Stm	***b*** = −0.79, *p* = 0.001*
Escalation: rapid burst episodes	M520/N	*b* = 1.35, *p* < 0.001*
	WKY/NCrl	*b* = 0.36, *p* = 0.098
	F344/NCrl	*b* = 0.70, *p* = 0.003*
	F344/Stm	*b* = 0.25, *p* = 0.094
	LEW/SS	*b* = 0.59, *p* = 0.059
	LEW/Crl	*b* = 0.30, *p* = 0.343
	LE/Stm	*b* = 0.58, *p* = 0.007*
Escalation: sustained burst episodes	M520/N	*b* = 0.98, *p* < 0.001*
	WKY/NCrl	*b* = 0.41, *p* = 0.030*
	F344/NCrl	*b* = 0.84, *p* < 0.001*
	F344/Stm	*b* = 0.50, *p* = 0.008*
	LEW/SS	*b* = 0.78, *p* = 0.004*
	LEW/Crl	*b* = 0.58, *p* = 0.066
	LE/Stm	*b* = 0.75, *p* < 0.001*

Significance indicated by emboldened text and asterisk (*p < 0.05).

In contrast, saline self-administration remained low throughout the escalation phase. Most strains exhibited progressive reductions in saline intake, corresponding to decreasing intake slopes over time (Supplementary Table 2). Although strain differences were detected early in the escalation phase (Strain X Session: *F*_54,__792_ = 3.287, *p* < 0.001), all strains reduced saline intake to comparable levels by LgA session 8 (Supplementary Figure 2). Analysis of the escalation index and the linear regression slop of saline intake failed to detect significant changes in saline intake (Supplementary Figure 2).

### 3.3 Strain-dependent dysregulation of oxycodone self-administration

**FIGURE 4:**
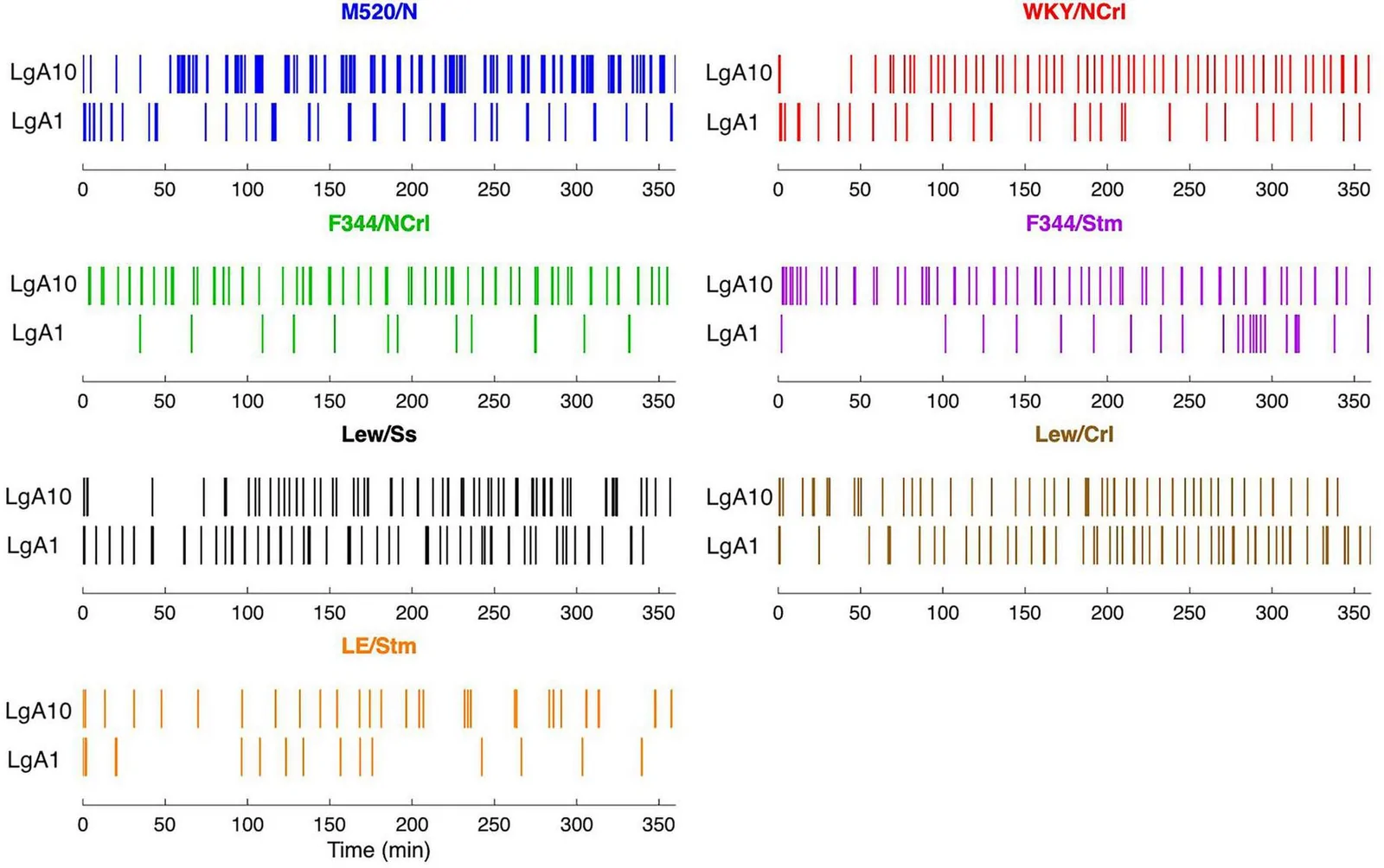
Representative within-session patterns of oxycodone intake during the escalation phase in multiple inbred rat strains. Raster plots show the timing of individual oxycodone infusions from representative animals of each strain during the 1st and 10th LgA sessions of the escalation phase.

To determine whether genetic background influences within-session patterns of oxycodone intake, we initially visualized raster plots of oxycodone self-administration across the escalation phase (Figure 4). We observed that some strains displayed progressively dysregulated oxycodone intake with high levels of burst-like responding characterized as clusters of closely spaced infusions separated by prolonged drug-free periods. To quantify the within-session patterns, we first analyzed inter-infusion intervals. Oxycodone intake escalation was accompanied by progressive shortening of inter-infusion intervals in nearly all strains, reflecting increasingly rapid within-session drug intake (Supplementary Figure 3). All strains except F344/Stm exhibited significant reductions in inter-infusion intervals across sessions (Table 3). Average inter-infusion intervals also differed among strains (*F*_6,__90_ = 3.19, *p* = 0.007), with F344/Stm rats exhibiting the longest inter-infusion intervals, consistent with their failure to escalate oxycodone intake.

**FIGURE 5:**
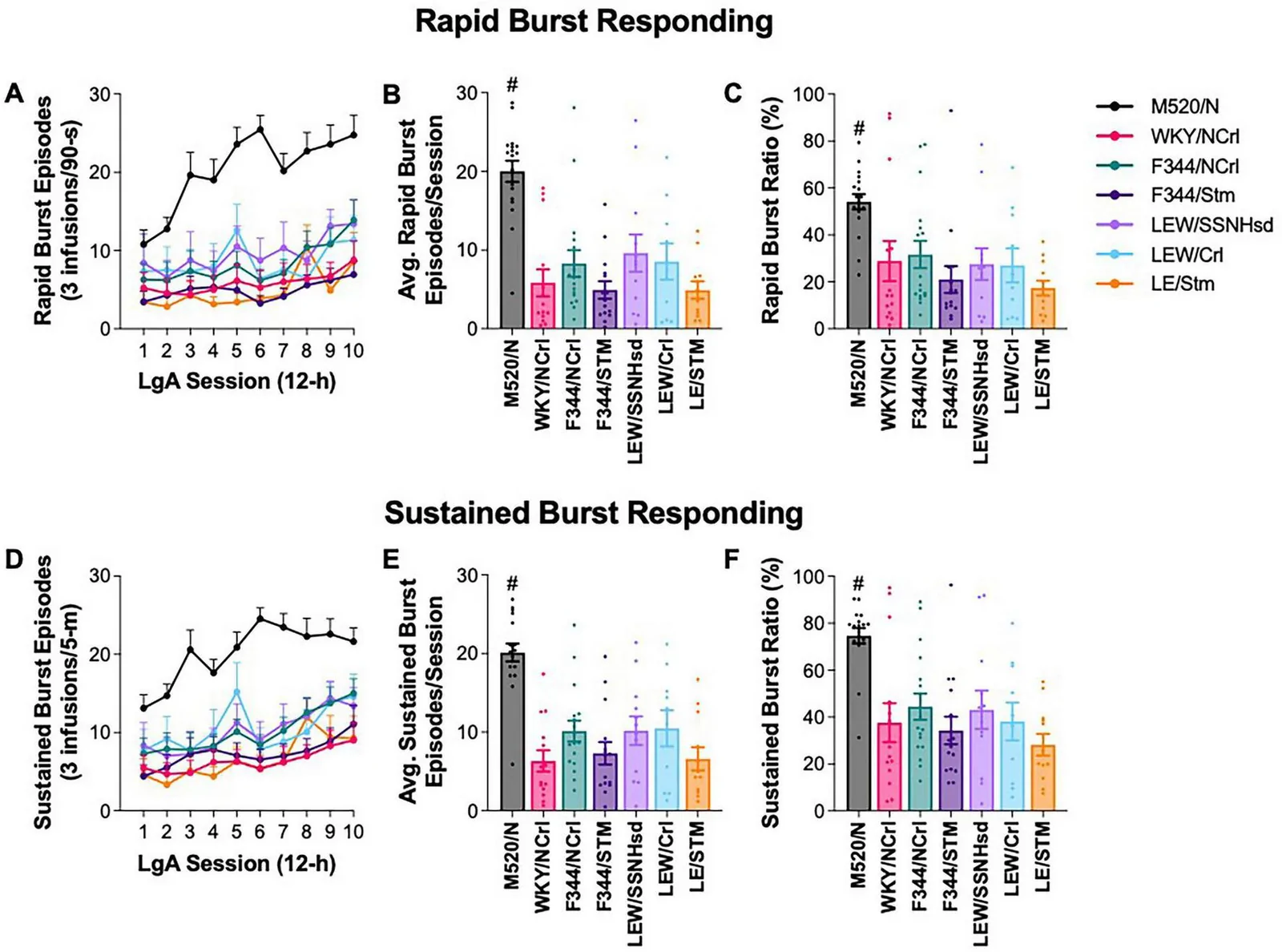
Strain differences in rapid and sustained burst responding during oxycodone self-administration. Two types of burst-like responding were recorded and analyzed across the ten 12-h long-access (LgA) sessions during the escalation phase. Rapid burst responding consists of brief, high-frequency clusters of infusions that terminate quickly. **(A)** Mean ( *SEM*) number of rapid burst episodes (minimally defined as 3 infusions within 90 s) across the escalation phase. A significant Strain x Session interaction indicated that all strains showed a progressive increase in rapid burst responding (*F*_14.75,__221.2_ = 1.86, *p* < 0.03). The M520/N strain exhibited markedly greater increases in rapid burst responding. **(B)** Analysis of the average number of rapid burst episodes per session across the escalation phase also revealed that the M520/N strain displayed greater overall rapid burst responding compared with all other strains (#*p* < 0.05). **(C)** The rapid burst ratio (%), represents the relative proportion of rapid burst responding relative to overall responding. The M520/N strain displayed a greater proportion of infusions delivered in rapid bursts compared with all other strains (#*p* < 0.05). Sustained bursts represented prolonged periods of continued high-rate drug intake. **(D)** Mean ( *SEM*) number of sustained burst episodes (minimally defined as 3 infusions within 5 min) across the escalation phase. Significant main effects of Strain (*F*_6,__90_ = 11.45, *p* < 0.001) and Session (*F*_2.543,__228.9_ = 15.66, *p* < 0.001) revealed that all strains increased responding in sustained bursts. **(E)** Analysis of the average number of sustained burst episodes per session across the escalation phase revealed that the M520/N strain displayed greater overall sustained burst responding compared with all other strains (#*p* < 0.05). **(F)** The sustained burst ratio (%) represents the relative proportion of sustained burst responding relative to overall responding. The M520/N strain displayed a greater proportion of infusions delivered in sustained bursts compared with all other strains (#*p* < 0.05). *N* = 10–17/strain.

Previous reports indicate that rats engage in dysregulated drug intake by self-administering short bursts of infusions followed by a long drug-free interval (Allain et al., 2015; Allain et al., 2017; Belin et al., 2009; Brown et al., 2022). Therefore, we next analyzed burst responding by determining two types of burst-like responding, rapid burst responding and sustained burst responding. Rapid burst responding consists of brief, high-frequency clusters of infusions that terminate quickly, whereas sustained bursts represented prolonged periods of continued high-rate drug intake. Strain differences were evident for both rapid and sustained burst responding (Figure 5). Mixed-effects modeling revealed a significant Strain x Session interaction indicating that while all strains showed a progressive increase in rapid burst responding, the M520/N strain exhibiting markedly greater increases in rapid burst responding (*F*_14.75,__221.2_ = 1.86, *p* < 0.03). Sustained burst responding also varied across Strain (*F*_6,__90_ = 11.45, *p* < 0.001) and Session (*F*_2.543,__228.9_ = 15.66, *p* < 0.001), with the M520/N strain showing overall greater sustained burst responding. Linear regression modeling revealed progressive increases in rapid burst responding for the M520/N, F344/NCrl, and LE/Stm strains, while all but the LEW/Crl strain displayed significant increases in sustained burst responding (Table 3). Although similar results were obtained with both types of burst responding, it is important to note that sustained burst responding represents a higher overall proportion of burst responding compared with rapid burst responding (Figures 5C,F). Nonetheless, these findings indicate that the M520/N strain displays an increased propensity for dysregulated oxycodone intake as indicted by both rapid and sustained burst responding. Burst responding was comparatively uncommon in saline self-administering rats (Supplementary Figure 4). Although modest session and strain differences were detected in rapid and sustained bursts, these effects revealed a progressive decrease in burst responding during the escalation phase and relatively minor strain differences compared to those observed during oxycodone self-administration.

**FIGURE 6:**
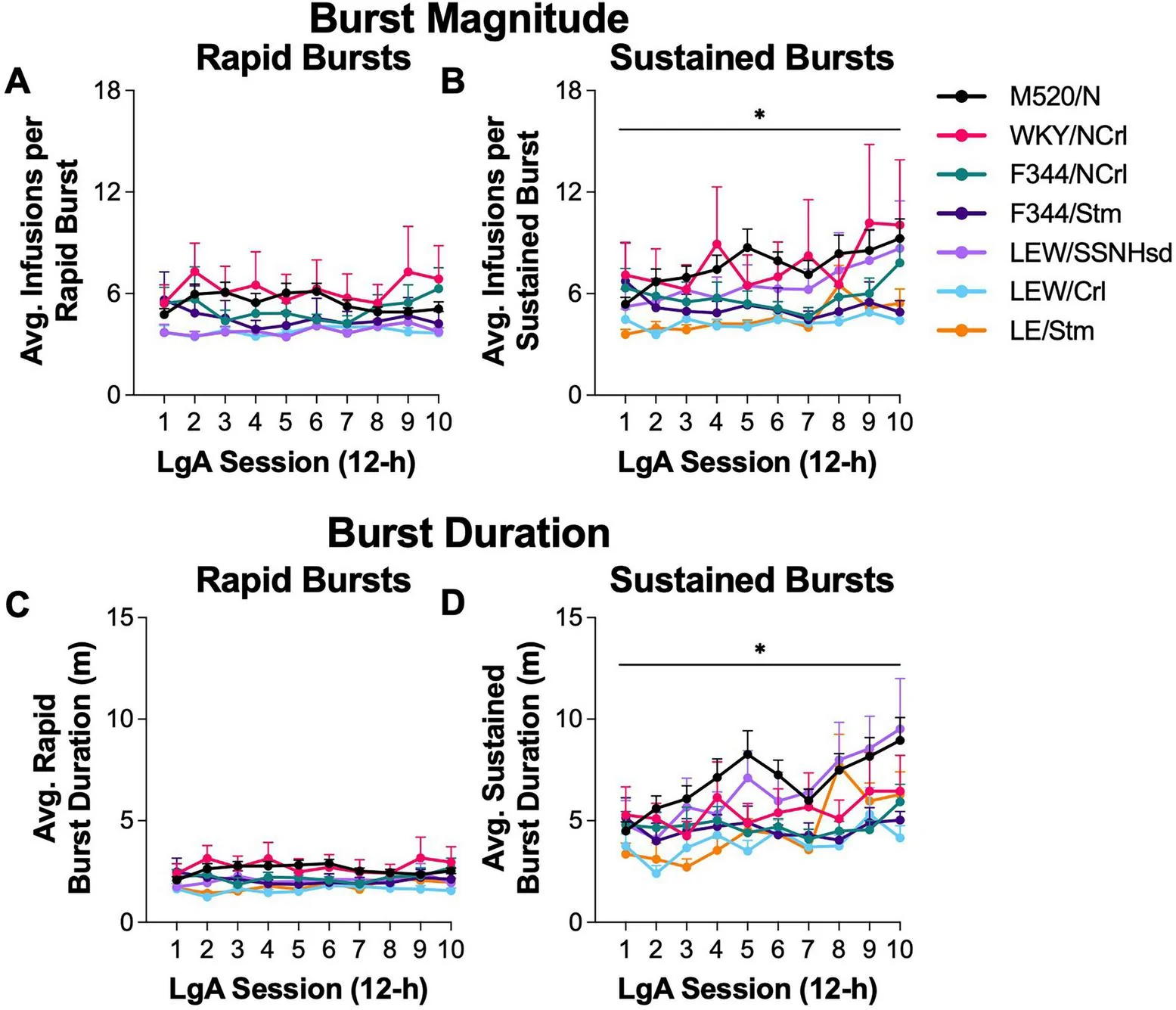
Characterization of burst magnitude and duration for rapid and sustained burst responding. Burst magnitude was calculated as the number of infusions within each rapid or sustained burst episode and averaged for each 12-h long-access (LgA) session. **(A)** The average rapid burst magnitude per session across escalation phase was remarkably consistent and did not vary between the strains. **(B)** The average sustained burst magnitude per session across the escalation phase significantly increased across all strains (*p < 0.05; main effect of session). The duration of rapid and sustained burst was also calculated for each LgA session. **(C)** The average rapid burst duration (m) showed little variability across the escalation phase and strains did not significantly differ. **(D)** The average sustained burst duration (m) of sustained burst episodes across LgA sessions displayed a significant main effect of Session (*F*_3.055,__244.4_ = 8.38, *p* < 0.001) indicating progressive increases across the escalation phase (*p < 0.05). A main effect of Strain was also observed, but no *post-hoc* comparisons survived correction (*F*_6,__90_ = 2.98, *p* = 0.01). *N* = 10–17/strain.

To further characterize burst behavior, we quantified number of infusions occurring within rapid and sustained burst episodes, the duration of rapid and sustained burst episodes, and the time between rapid and sustained bursts (Figure 6). Analysis of rapid burst magnitude and rapid burst duration indicated no significant differences between strains and demonstrated consistencies across the escalation phase. In contrast, both sustained burst magnitudes and sustained burst durations showed significant increases across the escalation phase (Sustained Burst Magnitude: *F*_2.021,__161.7_ = 3.24, *p* < 0.05; Sustained Burst Duration: *F*_3.055,__244.4_ = 8.38, *p* < 0.001). Sustained burst durations were also significantly different between the strains (*F*_6,__90_ = 2.98, *p* = 0.01). Together, these findings indicate that prolonged engagement in oxycodone-taking episodes may be a more sensitive behavioral feature that corresponds with oxycodone intake escalation and distinguishes genetically driven dysregulation phenotypes. Analysis of the temporal spacing between rapid and sustained burst episodes further supported these differences. The intervals between rapid bursts displayed subtle, but significant differences across strain with the F344/Stm and LE/Stm displaying the longest intervals between rapid bursts (Supplementary Figure 3; *F*_6,__90_ = 6.13, *p* < 0.001). Intervals between sustained bursts also displayed strain-dependent differences with the M520/N strain displaying the shortest intervals (Supplementary Figure 3; *F*_6,__90_ = 5.14, *p* < 0.001).

To identify whether strains displaying high levels of burst responding also displayed high non-specific motoric activity or indiscriminative responding, we evaluated inactive lever responses and timeout responses across the escalation phase (Supplementary Figure 5). Mixed-effects models revealed a significant main effect of Strain (*F*_6,__90_ = 2.40, *p* = 0.033), although no pairwise strain comparisons survived multiple comparison correction. Analyses of timeout responding revealed no significant differences between the strains. Together these findings suggest that high burst responding observed in the M520/N strain is not inherently accompanied by non-specific motoric activity, indiscriminative responding, or perseverative behavior during timeout periods.

### 3.4 Relationships between acquisition, escalation, and dysregulation phenotypes

**FIGURE 7:**
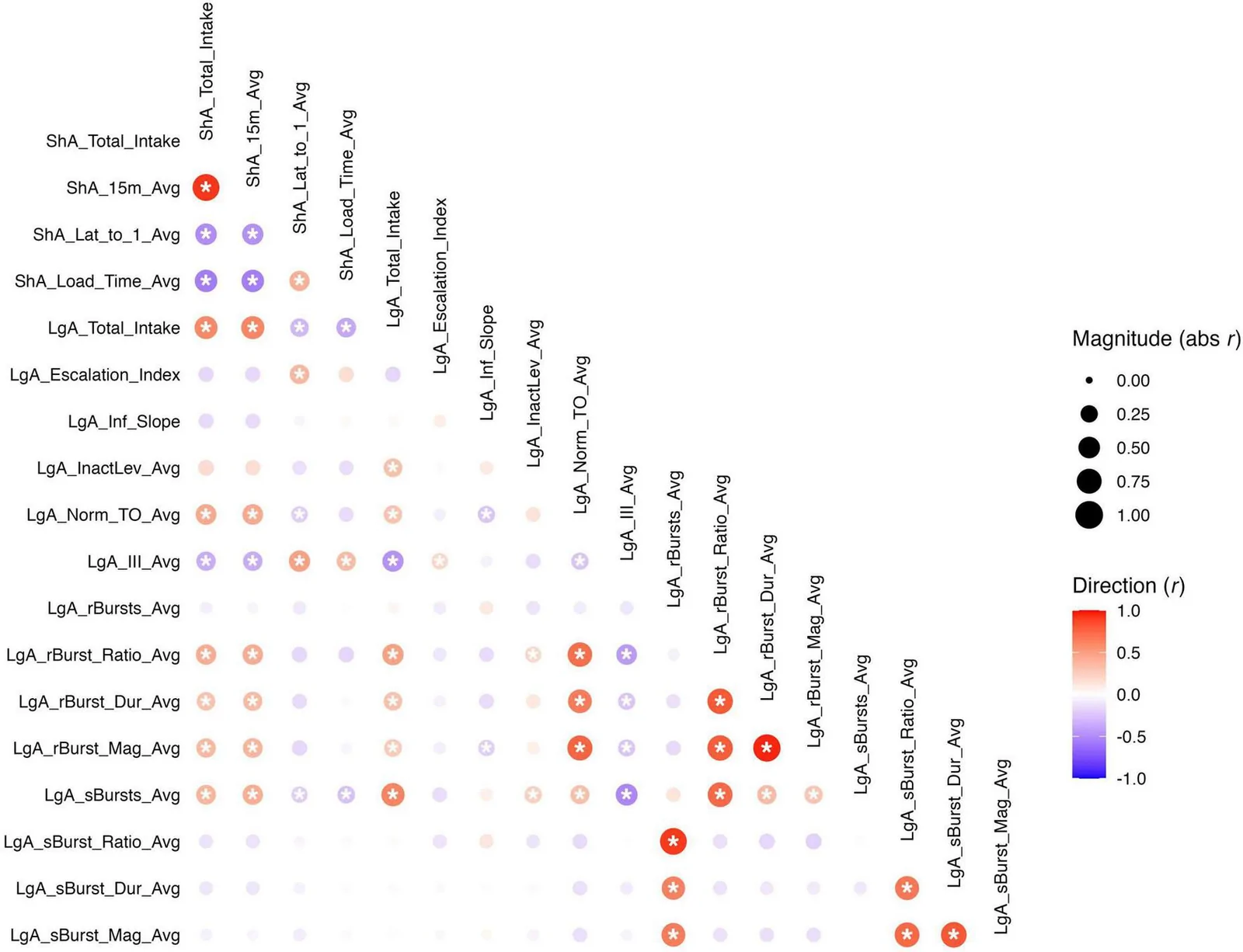
Correlation matrix of oxycodone acquisition, escalation, and dysregulated self-administration behaviors. Pearson correlations were calculated across individual rats from seven inbred rat strains (e.g., M520/N, WKY/NCrl, F344/NCrl, F344/Stm, LEW/SSNHsd, LEW/Crl, and LE/Stm). The correlation matrix illustrates the relationships among key behavioral metrics across short-access (ShA) acquisition, long-access (LgA) escalation, and burst-responding dynamics during oxycodone self-administration. Circle size represents the magnitude of the absolute correlation coefficient (abs r) Circle color indicates the direction and strength of the correlation (r) ranging from strong negative (blue, −1.0) to neutral (white, 0.0) to strong positive (red, +1.0). White asterisks inside circles denote statistically significant correlations (*p*0.05). ShA, Short-Access; LgA, Long-Access; Total_Intake, cumulative drug intake (mg/kg); 15 m_Avg, average infusions during the first 15 min; Lat_to_1_Avg, average latency to 1st infusion; Load_Time_Avg, average drug load time; Escalation_Index, measure of LgA intake escalation relative to baseline; Inf_Slope, linear regression slope of LgA infusions; InactLev_Avg, average LgA inactive lever presses; Norm_TO_Avg, LgA normalized time-out responding; rBurst, rapid burst responding; sBurst, sustained burst responding; Dur, duration (m); Mag, magnitude (infusions per burst episode). *p < 0.05, *N* = 10/17/strain; Total *N* = 97.

Correlation analyses were performed to examine the relationships among phenotypes within specific behavioral domains including initiation of oxycodone intake (i.e., acquisition), escalation of oxycodone intake, and indices of dysregulated oxycodone intake, including rapid and sustained burst responding (Figure 7). Overall, the correlation matrix revealed distinct clusters of related behavioral measures, indicating that acquisition, escalation, and dysregulation represent overlapping but separable components of oxycodone self-administration behavior. For example, during the acquisition phase, both total oxycodone intake and oxycodone intake during the first 15-m of the ShA session were positively associated with oxycodone intake during the escalation phase. Interestingly, oxycodone intake during the acquisition phase also displayed modest positive correlations with burst responding during the escalation phase. These findings suggest that rats exhibiting greater initial drug taking are more likely to maintain higher levels of oxycodone intake during escalation and are more likely to display dysregulated oxycodone intake. In contrast, latency-based measures during acquisition (e.g., Latency to 1st infusion and Load Time) were generally inversely related to intake, indicating that rapid initiation of oxycodone self-administration predicted greater overall intake.

During the escalation phase, total oxycodone intake, escalation indices, and measures reflecting patterns of drug consumption showed moderate positive correlations, demonstrating that increased intake was accompanied by progressively altered patterns of self-administration. Dysregulation metrics based on burst responding also exhibited clear clustering. Within both the rapid burst (rBurst) and sustained burst (sBurst) domains, measures describing burst frequency, duration, magnitude, and burst ratio were strongly intercorrelated, supporting the internal consistency of each behavioral construct. However, correlations between rapid and sustained burst measures were surprisingly modest, suggesting that the two approaches capture complementary aspects of dysregulated drug-taking behavior. This was also evident in how these two approaches correlate with other measures. For example, normalized timeout responding during escalation exhibited strong positive correlations with several rapid burst metrics, including the rapid burst ratio, rapid burst duration, and rapid burst magnitude. In contrast, normalized timeout responding showed comparatively weak or absent correlations with the sustained burst metrics. These relationships indicate that rats displaying high levels of timeout responding also engage in impulsive, high-frequency bouts of oxycodone intake than with more prolonged periods of intense oxycodone intake. These findings further support the interpretation that rapid and sustained burst analyses capture distinct facets of dysregulated drug-taking behavior, and that conventional measures of drug intake and escalation are associated with but do not fully explain the temporal organization of oxycodone self-administration.

### 3.5 Sex differences

Mixed-effect models were utilized to determine if sex differences were present in several acquisition measures across the ShA sessions including total infusions, infusions in the first 15-m of the session, latency to the 1st infusion, and load time (Supplementary Figure 7). Here, only rats in the oxycodone group were analyzed and we only report significant main effects of Sex or the interaction between Sex and Session. No significant main effect of Sex or Sex-Session interaction was observed in total oxycodone infusions during acquisition, although WKY/NCrl males displayed a trend toward greater intake compared with females (*F*_1,__12_ = 3.48, *p* = 0.087). Regression analyses demonstrated significant increases in oxycodone intake across acquisition in both sexes for nearly all strains, with WKY/NCrl males exhibiting a significantly steeper acquisition slope than females (Table 4). Analysis of early session responding revealed no significant Sex or Sex × Session effects, although strain-specific differences in acquisition rates were observed using regression modeling. Female M520/N and F344/NCrl rats displayed steeper increases in early session infusions compared with males, whereas male LEW/SS and LE/Stm rats showed steeper increases than females (Table 4). Sex differences were also observed in latency measures with male M520/N rats displaying more rapid oxycodone intake initiation, particularly during the initial acquisition sessions (Sex × Session: *F*_9,__135_ = 2.59, *p* = 0.009). Male LEW/SS rats, on the other hand, exhibited longer first-infusion latencies during the first session (Sex × Session: *F*_9,__90_ = 2.74, *p* = 0.007). Regression analyses confirmed these effects, with females showing steeper decreases in latency in M520/N rats and males showing steeper decreases in LEW/SS rats (Table 4). Load time analysis further identified sex-specific differences in M520/N and F344/NCrl rats, with females displaying longer load times across acquisition (M520/N: *F*_9,__135_ = 3.74, *p* < 0.001; F344/NCrl: *F*_9,__62_ = 2.24, *p* = 0.031). Overall, sex differences during acquisition were relatively subtle and were primarily evident in measures of response initiation, with M520/N and WKY/NCrl males generally exhibiting faster engagement with oxycodone reinforcement compared with females.

**TABLE 4:** Linear regression analyses for males and females in the oxycodone self-administration group.

Measure	Strain	Male	Female	Male v. female *F*-test
Acquisition: infusions	M520/N	*b* = 0.70, *p* = 0.002*	*b* = 1.47, *p* < 0.001*	*F*_1,__166_ = 3.24, *p* = 0.074
	WKY/NCrl	*b* = 1.27, *p* < 0.001*	*b* = 0.57, *p* = 0.007*	*F*_1,__136_ = 4.65, *p* = 0.033*
	F344/NCrl	*b* = 1.80, *p* < 0.001*	*b* = 2.21, *p* < 0.001*	*F*_1,__166_ = 0.57, *p* = 0.451
	F344/Stm	*b* = 0.96, *p* = 0.164	*b* = 0.77, *p* = 0.007*	*F*_1,146_ = 0.04, *p* = 0.844
	LEW/SS	*b* = 1.17, *p* = 0.028*	*b* = 0.20, *p* = 0.633	*F*_1,__116_ = 2.14, *p* = 0.146
	LEW/Crl	*b* = 0.21, *p* = 0.706	*b* = 0.40, *p* = 0.270	*F*_1,__96_ = 0.09, *p* = 0.770
	LE/Stm	*b* = 1.38, *p* < 0.001*	*b* = 0.82, *p* = 0.003*	*F*_1,__116_ = 1.58, *p* = 0.212
Acquisition: 15 min infusions	M520/N	*b* = 0.28, *p* = 0.005*	*b* = 0.65, *p* < 0.001*	*F*_1,__166_ = 5.50, *p* = 0.020*
	WKY/NCrl	*b* = 0.33, *p* < 0.001*	*b* = 0.21, *p* = 0.001*	*F*_1,136_ = 1.29, *p* = 0.259
	F344/NCrl	*b* = 0.28, *p* < 0.001*	*b* = 0.56, *p* < 0.001*	*F*_1,__166_ = 5.29, *p* = 0.023*
	F344/Stm	*b* = 0.45, *p* = 0.032*	*b* = 0.22, *p* = 0.020*	*F*_1,__146_ = 0.60, *p* = 0.439
	LEW/SS	*b* = 0.40, *p* = 0.001*	*b* = 0.05, *p* = 0.637	F_1,116_ = 5.46, *p* = 0.021*
	LEW/Crl	*b* = 0.05, *p* = 0.695	*b* = 0.16, *p* = 0.079	*F*_1,__96_ = 0.48, *p* = 0.492
	LE/Stm	*b* = 0.57, *p* < 0.001*	*b* = 0.23, *p* = 0.008*	*F*_1,__116_ = 5.32, *p* = 0.023*
Acquisition: latency to 1st infusion	M520/N	***b*** = −0.39, *p* = 0.005*	*b* = −4.33, *p* < 0.001	*F*_1,__166_ = 17.20, *p* < 0.001*
	WKY/NCrl	*b* = −1.39, *p* = 0.062	***b*** = −2.66, *p* = 0.025*	*F*_1,__136_ = 0.03, *p* = 0.872
	F344/NCrl	*b* = −0.65, *p* = 0.427	***b*** = −4.26, *p* = 0.016*	*F*_1,__166_ = 3.83, *p* = 0.052
	F344/Stm	***b*** = −4.13, *p* = 0.010*	***b*** = −6.37, *p* = 0.021*	*F*_1,__146_ = 0.59, *p* = 0.443
	LEW/SS	***b*** = −1.74, *p* = 0.009*	*b* = −0.05, *p* = 0.929	*F*_1,__116_ = 4.45, *p* = 0.037*
	LEW/Crl	***b*** = −0.16, *p* = 0.005*	*b* = −0.23, *p* = 0.063	*F*_1,__96_ = 0.27, *p* = 0.604
	LE/Stm	*b* = −5.12, *p* = 0.017	*b* = −1.24, *p* = 0.540	*F*_1,__116_ = 1.47, *p* = 0.227
Acquisition: load time	M520/N	*b* = −0.11, *p* = 0.361	***b*** = −5.08, *p* < 0.001*	*F*_1,__166_ = 16.80, *p* < 0.001*
	WKY/NCrl	***b*** = −4.07, *p* = 0.004*	*b* = −3.61, *p* = 0.014	*F*_1,__136_ = 0.06, *p* = 0.815
	F344/NCrl	*b* = −1.85, *p* = 0.133	*b* = −7.05, *p* < 0.001	*F*_1,__166_ = 6.70, *p* = 0.011*
	F344/Stm	***b*** = −3.96, *p* = 0.025*	***b*** = −7.74, *p* = 0.003*	*F*_1,__146_ = 1.56, *p* = 0.214
	LEW/SS	*b* = −0.49, *p* = 0.215	*b* = −0.19, *p* = 0.871	*F*_1,__116_ = 0.30, *p* = 0.582
	LEW/Crl	*b* = −0.54, *p* = 0.285	*b* = −0.45, *p* = 0.251	*F*_1,__96_ = 0.02, *p* = 0.882
	LE/Stm	***b*** = −8.37, *p* = 0.001*	*b* = −4.61, *p* = 0.030	*F*_1,__116_ = 1.23, *p* = 0.270
Escalation: Infusions	M520/N	*b* = 6.13, *p* = 0.018*	*b* = 9.07, *p* < 0.001*	*F*_1,__166_ = 0.78, *p* = 0.379
	WKY/NCrl	*b* = 6.93, *p* = 0.056	*b* = 4.635, *p* < 0.001*	*F*_1,__136_ = 0.38, *p* = 0.537
	F344/NCrl	*b* = 4.35, *p* < 0.001*	*b* = 8.81, *p* = 0.005*	*F*_1,__166_ = 2.03, *p* = 0.156
	F344/Stm	*b* = 3.70, *p* = 0.114	*b* = 2.18, *p* = 0.316	*F*_1,__116_ = 0.18, *p* = 0.676
	LEW/SS	*b* = 3.05, *p* = 0.327	*b* = 11.83, *p* < 0.001*	*F*_1,__116_ = 4.28, *p* = 0.041*
	LEW/Crl	*b* = 4.73, *p* = 0.061	*b* = 7.88, *p* < 0.001*	*F*_1,__96_ = 1.03, *p* = 0.312
	LE/Stm	*b* = 9.33, *p* < 0.001*	*b* = 9.37, *p* < 0.001*	*F*_1,__116_ = 0.00, *p* = 0.991
Escalation: rapid burst episodes	M520/N	*b* = 0.87, *p* = 0.019*	*b* = 1.77, *p* < 0.001*	*F*_1,__166_ = 3.61, *p* = 0.059
	WKY/NCrl	*b* = 0.23, *p* = 0.518	*b* = 0.48, *p* = 0.003*	*F*_1,__136_ = 0.40, *p* = 0.526
	F344/NCrl	*b* = 0.59, *p* = 0.001*	*b* = 0.82, *p* = 0.054	*F*_1,__166_ = 0.27, *p* = 0.602
	F344/Stm	*b* = 0.09, *p* = 0.656	*b* = 0.58, *p* = 0.010*	*F*_1,__146_ = 2.44, *p* = 0.121
	LEW/SS	*b* = 0.10, *p* = 0.807	*b* = 1.08, *p* = 0.021*	*F*_1,__116_ = 2.55, *p* = 0.113
	LEW/Crl	*b* = 0.01, *p* = 0.981	*b* = 0.58, *p* = 0.133	*F*_1,__96_ = 0.87, *p* = 0.353
	LE/Stm	*b* = 0.87, *p* = 0.002*	*b* = 0.44, *p* = 0.135	*F*_1,__116_ = 0.90, *p* = 0.346
Escalation: Sustained Burst Episodes	M520/N	*b* = 0.92, *p* = 0.010*	*b* = 1.31, *p* < 0.001*	*F*_1,__176_ = 0.91, *p* = 0.342
	WKY/NCrl	*b* = 0.26, *p* = 0.392	*b* = 0.55, *p* = 0.004*	*F*_1,__136_ = 0.63, *p* = 0.429
	F344/NCrl	*b* = 0.70, *p* = 0.002*	*b* = 1.00, *p* = 0.003*	*F*_1,__166_ = 0.61, *p* = 0.436
	F344/Stm	*b* = 0.33, *p* = 0.150	*b* = 0.84, *p* = 0.012*	*F*_1,__146_ = 1.64, *p* = 0.203
	LEW/SS	*b* = 0.13, *p* = 0.738	*b* = 1.43, *p* < 0.001*	*F*_1,__116_ = 6.32, *p* = 0.013*
	LEW/Crl	*b* = 0.17, *p* = 0.714	*b* = 0.98, *p* = 0.012*	*F*_1,__96_ = 1.86, *p* = 0.176
	LE/Stm	*b* = 1.11, *p* = 0.002*	*b* = 0.57, *p* = 0.050*	*F*_1,__116_ = 1.35, *p* = 0.248

Significance indicated by emboldened text and asterisk (*p < 0.05).

**FIGURE 8:**
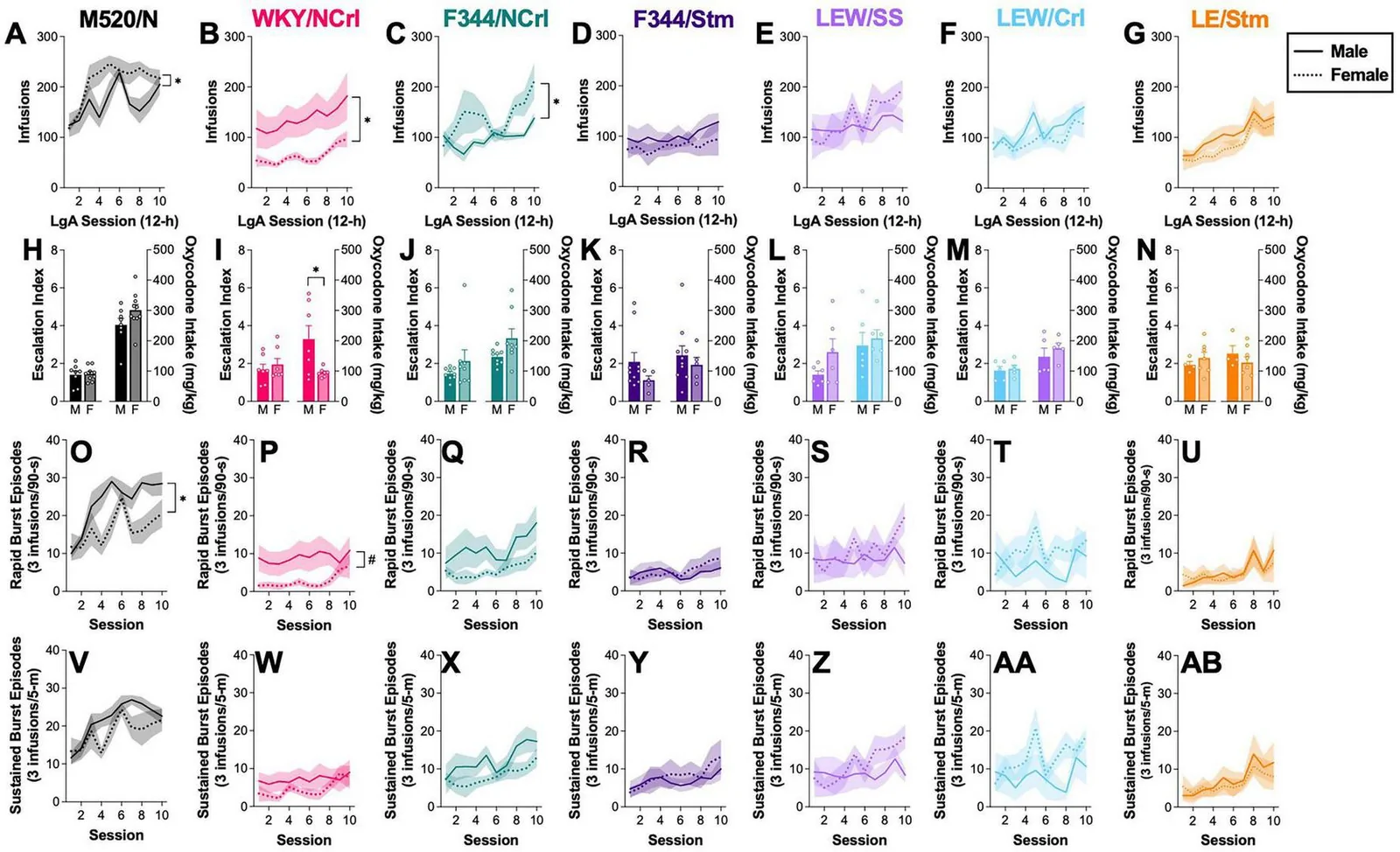
Comparison between male and females in escalation metrics. Total infusions per 12-h session during the escalation phase is displayed for males (solid line) and females (dashed line) in the **(A)** M520/N (*N* = 8M/9F), **(B)** WKY/NCrl (*N* = 7M/7F), **(C)** F344/NCrl (*N* = 9M/8F), **(D)** F344/Stm (*N* = 10M/5F), **(E)** LEW/SS (*N* = 6M/6F), **(F)** LEW/Crl (*N* = 5M/5F), and **(G)** LE/Stm (*N* = 4M/8F) strains. M520/N and F344/NCrl females displayed greater oxycodone intake compared with M520/N and F344/NCrl females, while WKY/NCrl males displayed greater oxycodone intake compared with WKY/NCrl males (*p < 0.05). The escalation index (left) and total oxycodone intake (right) during the escalation phase is displayed for males (dark bar) and females (light bar) in **(H–N)**. No sex differences were observed in the escalation index for any strains. The WKY/NCrl males displayed significantly more total oxycodone intake compared with females (*p < 0.05). The number of rapid burst episodes during each session is displayed for males (solid line) and females (dashed line) in **(O–U)**. Female M520/N rats displayed significantly greater burst responding compared with females (*p < 0.05) while male WKY/NCrl rats trended toward greater burst responding (#*p* = 0.07). All data shown as mean ± SEM. The number of sustained burst episodes during each session is displayed for males (solid line) and females (dashed line) in **(V-AB)**. No significant sex differences were observed using a mixed effect model for each strain.

Mixed-effect models were also used to determine if sex differences were present in the escalation and dysregulation of oxycodone intake (Figure 8). Again, only significant main effects of Sex or the interaction between Sex and Session are reported. A significant main effect of Sex was observed for the WKY/NCrl strain in the total number of oxycodone infusions per session with males having higher intake compared with females (*F*_1,__12_ = 6.11, *p* = 0.029). A significant Sex-Session interaction was observed for the M520/N, F344/NCrl, and LEW/SS strains with higher intake observed in female rats on several of the LgA sessions (M520/N: *F*_9_, _135_ = 1.98, *p* = 0.047; F344/NCrl: *F*_9_, _135_ = 2.94, *p* = 0.003; LEW/LEW/SS: *F*_9,__90_ = 2.03, *p* = 0.044). Regression modeling indicated robust positive slopes in both males and females in nearly all strains with female LEW/SS rats displaying a significantly steeper slope than male LEW/SS rats (Table 4). Using an unpaired *t*-test to compare the escalation index between males and females revealed no sex differences. Analysis of total oxycodone intake between males and females of each strain indicated higher intake in male WKY/NCrl rats compared with females (*t*_6.19_ = 2.47, *p* = 0.047), but no other strains. Analysis of the number of rapid burst episodes during escalation revealed that female M520/N rats displayed significantly more rapid burst episodes than males (*F*_1,__15_ = 10.01, *p* = 0.006). In contrast, male WKY/NCrl rats had a higher number of rapid burst episodes than females that trended toward significance (*F*_1,__12_ = 3.99, *p* = 0.069). Mixed effects models revealed no sex differences in sustained burst responding for any of the strains tested. Only the LEW/SS strain displayed a significant difference between males and females using regression modeling indicating that females displayed greater change in sustained burst responding compared to males to analyze rapid and sustained burst responding (Table 4). Considering that male M520/N rats displayed faster early session measures while female M520/N rats displayed a higher number of infusions and burst episodes during escalation, this suggests that distinct biological systems may govern the acquisition of oxycodone self-administration and dysregulation of oxycodone self-administration.

## 4 Discussion

The use of inbred rat strains enables the identification of genetic contributions to specific OUD-related phenotypes that are difficult to detect in human studies. We analyzed a subset of inbred rat strains that displayed robust oxycodone intake escalation from our prior studies using a larger panel of inbred strains (Duffy et al., 2024a). We found that strains differed in the acquisition of oxycodone self-administration suggesting that genetic variability may influence the initial reinforcing effects of oxycodone. There were also significant strain differences in early session latency measures during acquisition supporting differences in the motivation and reinforcement learning associated with early self-administration of oxycodone. While all strains displayed an increase in oxycodone intake during the escalation phase, the M520/N strain self-administered significantly more oxycodone than all other tested strains. Moreover, the M520/N strain displayed a high rate of dysregulated oxycodone intake as measured by burst responding, a pattern of self-administration where several oxycodone self-infusions are delivered in a short period of time. Our findings also demonstrate that burst responding is not a unitary behavior but consists of at least two complementary patterns of oxycodone intake. By separating burst responding into rapid and sustained bursts, we revealed that these measures capture distinct aspects of dysregulated oxycodone intake. Whereas rapid bursts remained relatively stable throughout escalation, sustained bursts progressively increased in duration and magnitude, suggesting that prolonged episodes of drug taking may represent a more sensitive behavioral marker of escalating dysregulation. The M520/N strain exhibited elevations in both forms of burst responding, further supporting its value as a model of genetically driven vulnerability to opioid misuse. These findings suggest that genetic factors contribute to distinct behavioral phenotypes, including within-session regulation of drug intake that may reflect the dysregulated and impaired control over oxycodone use.

In a study utilizing a similar IVSA paradigm, Doyle et al. (2023) examined burst responding, defined as a train of drug infusions that continues if there is < 90 s between infusions, in several inbred strains including the same WKY/NCrl and F344/NCrl strains assessed here. Consistent with our findings, they observed approximately 30% burst responding in the WKY/NCrl and F344/NCrl strains and similar within-session distribution of oxycodone infusions as measured by inter-infusion intervals. We demonstrate for the first time that the M520/N strain displays a striking pattern of oxycodone self-administration. In addition to showing relatively fast acquisition metrics, and high overall oxycodone intake during escalation, the M520/N strain engaged in significantly more burst responding with a significantly higher proportion of infusions (50–80%) occurring within bursts compared with other strains. Interestingly, female M520/N rats displayed a greater propensity to engage in rapid burst responding compared with male M520/N rats suggesting that even within a genetically vulnerable strain, sex differences contribute to the microstructure of drug-taking behavior and may reflect distinct underlying motivational or neurobiological mechanisms driving opioid intake.

Our correlation analyses highlight that acquisition, escalation, and dysregulated oxycodone intake represent related but distinct behavioral phenotypes. For example, initial responding for oxycodone is associated with later dysregulated drug use as indicated by several acquisition metrics displaying significant correlation coefficients with oxycodone intake and burst responding during the escalation phase. We also identified that rapid and sustained burst metrics formed distinct correlation clusters, indicating that they capture complementary aspects of oxycodone self-administration. For example, rapid burst measures were strongly associated with timeout responding, suggesting that rapid bursts reflect short bouts of intense drug seeking despite reinforcement unavailability. Sustained burst measures, on the other hand, showed weaker relationships with timeout responding suggesting that they may represent prolonged episodes of excessive drug consumption. Importantly, these associations were evident at the individual-animal level rather than between strains. Thus, the M520/N strain exhibited robust burst responding, but did not display elevated inactive lever or timeout responding. This finding indicates that burst responding is unlikely to reflect generalized hyperactivity or perseverative responding. Together, these findings demonstrate that analyses of behavioral microstructure reveal individual variation in oxycodone intake regulation that may not be captured by strain-level comparisons or traditional measures of total drug intake.

The bursting pattern of intake displayed in some rats mirrors several features of human drug consumption that have been modeled in the intermittent access procedure. In this procedure, rats have short periods of drug access separated by longer intervals of abstinence (Zimmer et al., 2012). Intermittent access models are thought to capture cyclical patterns of intense drug-taking in humans (Kuhn et al., 2019). Some strains, particularly the M520/N strain, appear to naturally engage in a pattern of oxycodone use that is reflective of short bouts of intense drug-taking, making this a potentially clinically relevant phenotype. Although the limited access procedure was developed to mimic psychostimulant drug use, recent studies have incorporated intermittent access to heroin and oxycodone and found that it enhances drug craving throughout abstinence (D’Ottavio et al., 2023; Samson et al., 2022). Interestingly, burst-like responding to heroin self-administration was observed in both an intermittent-access model and a continuous-access model that removes the timeout period following heroin infusions in outbred Sprague Dawley rats (D’Ottavio et al., 2025a). Burst responding is reminiscent of binge-like drug use, typically defined as intense drug consumption in a limited temporal window followed by a period of abstinence (Beacher et al., 2025). Binge drug use is associated with increased risk for overdose (Kerr et al., 2007). While binge patterns of opioid use are less reported in clinical studies, a survey of intravenous drug-users in Montreal found that binge use of opioids was observed in nearly 20% of all drug binge episodes with binge use of opioid/stimulant combinations occurring in approximately 14% of binge episodes (Minoyan et al., 2025). Although the specific temporal criteria for a binge differs depending on the drug class and pharmacokinetic profile, burst responding may reflect binge-like use during a single drug-taking session (Brown et al., 2022; Koob and Volkow, 2010).

The inability to regulate drug intake through binge patterns of use has been associated with the development of addiction-related behaviors such as increased motivation, intake escalation, and relapse to drug seeking (Allain et al., 2015; Allain et al., 2017; Kawa et al., 2016). For example, burst responding was associated with a higher preference for heroin compared to social reward in a drug-versus-social choice procedure in Sprague-Dawley rats (D’Ottavio et al., 2025b). Moreover, the preference for heroin over social interaction positively correlated with burst-like heroin self-administration, suggesting that burst-like responding predicts reduced responsiveness to alternative rewards and aberrant drug motivation. It would be interesting to identify if the M520/N strain demonstrates similar increased drug motivation and reduced motivation for social interaction. Other work in Sprague-Dawley rats demonstrated that temporal distribution of oxycodone infusions during escalation predicted subsequent reinstatement to oxycodone seeking in a sex-dependent manner. These findings suggest that the microstructural organization of intake may be a critical determinant of relapse risk (Guha et al., 2022). Together, these findings indicate that some strains, such as the M520/N strain, may provide useful genetic models for identifying the genes and biological mechanisms that promote dysregulated, binge-like opioid intake, and other potentially related phenotypes such as impulsivity, metabolism of oxycodone, and drug-induced plasticity that leads to OUD.

A recent study utilized an oral oxycodone self-administration paradigm to examine strain variation in oral oxycodone intake across 35 inbred rat strains (Sharp et al., 2024). Interestingly, the M520/N strain displayed relatively low oral intake of oxycodone compared with the other strains tested. This contrasts with our observation that M520/N rats rapidly escalate intravenous oxycodone intake, display high burst responding, and have high overall oxycodone intake. It is plausible that oral and IVSA paradigms model partially overlapping but distinct components of drug reinforcement and intake patterns, and divergent intake patterns between the two models could be due to a variety of factors. For example, the metabolism and pharmacokinetics of oxycodone differ significantly between oral and intravenous procedures where potential first-pass elimination and delayed drug onset could impact its reinforcing effects when consumed orally (Kalso, 2005; Leow et al., 1992). Oral procedures are also sensitive to palatability differences that may be more prevalent in some strains (Tordoff et al., 2008). Furthermore, the M520/N strain displayed high oral intake of alcohol compared with other inbred strains, even rat lines selectively bred for high alcohol consumption (Ting-Kai and Lumeng, 1984). Direct assessment of M520/N oxycodone metabolism across these routes of administration, as well as taste reactivity experiments, will shed light on the intake discrepancies observed between these procedures.

The Fischer (F344) and Lewis strains have been used as a genetic model of resistance and susceptibility, respectively, to substance use disorders, including OUD (Cadoni, 2016). Prior work found that the Lewis strain acquires heroin and morphine self-administration faster, display higher responding for both opioids under various opioid reinforcement schedules, and escalate intake at their preferred dose of heroin compared to the F344 strain (Ambrosio et al., 1995; Martı’n et al., 1999; Picetti et al., 2012; Sánchez-Cardoso et al., 2007). Mechanistically, strain differences in mu opioid receptor affinity and lower expression of endogenous opioids in the Lewis strain suggest a reduced basal tone of opioids (Herradón et al., 2003; Martı’n et al., 1999). Comparisons of female LEW/Hsd and F344/Hsd rats in an oral oxycodone support the divergent intake patterns with LEW/Hsd females consuming more oxycodone, especially at higher oxycodone concentrations (Sharp et al., 2021). More recent findings suggest more nuanced differences between the F344 and Lewis strains that is highly dependent on the substrains (Sharp et al., 2024). We did not observe major differences in intravenous oxycodone intake between two F344 substrains (F344/NCrl and F344/Stm) and two Lewis substrains (LEW/Crl and LEW/SSNHsd). The Lewis and F344 substrains displayed similar oxycodone intake patterns with differences between the two strains in the early session metrics during acquisition where both Lewis substrains displayed significantly faster latencies to the first infusion and faster load times. This supports prior observations that the Lewis strain develops self-administration behavior more readily than the F344 strain. There were also subtle differences between the F344/NCrl and F344/Stm substrains in the inter-infusion intervals during the escalation phase. Differences between substrain behaviors can provide valuable insights into slight genetic divergences that could be useful in identifying genetic variants and establishing more refined genetic models (Abdurahaman et al., 2025; Beierle et al., 2022; Bryant et al., 2008; Kantak et al., 2021; Kirkpatrick and Bryant, 2015). Together, these findings indicate that phenotypic differences between the Lewis and F344 strains are nuanced and substrain-dependent, underscoring the importance of genetic resolution when modeling vulnerability and resistance to opioid use disorder.

Male and female rats of each strain were compared using acquisition, escalation, and burst-related phenotypes. Analysis of the early session measures during the acquisition phase revealed sex differences in a subset of the tested strains. Of note, male M520/N rats displayed faster latencies to the first infusion and load times than female M520/N rats, while male F344/NCrl rats displayed faster load times compared with females. These differences were most apparent during the first few ShA acquisition sessions suggesting that males may engage in greater exploratory responding during the early portions of these sessions. Sex differences in the escalation measures were observed in the M520/N, WKY/NCrl, and F344/NCrl strains. Aligning with load time differences, male WKY/NCrl rats consumed significantly more oxycodone than female WKY/NCrl rats. In contrast, female M520/N and F344/NCrl rats displayed greater responding for oxycodone throughout the escalation phase. A recent study reported no differences between male and female oxycodone intake in the WKY/N and F344/N strains, despite males showing higher peak concentrations in plasma oxycodone (Doyle et al., 2023). Prior work in the M520/N strain did not reveal sex differences in an oral oxycodone procedure, suggesting the differences observed in the present study may be specific to intravenous oxycodone self-administration (Sharp et al., 2024). In the same study, female LE/Stm rats consumed more oral oxycodone compared with male LE/Stm rats, a sex-dependent effect that was not observed in the present study (Sharp et al., 2024). In an earlier oral oxycodone study, it was reported that female LEW/Hsd rats consumed significantly more oxycodone that male LEW/Hsd rats (Sharp et al., 2021). Although we did not see differences in overall oxycodone intake in either Lewis strain, the LEW/SS females displayed a steeper slope during the escalation phase indicating a more rapid escalation of intake. Sex differences in the number of burst episodes during escalation sessions were only found in the M520/N strain, with females engaging in more burst responding than males. In a previous report, inter-infusion intervals and inter-burst intervals were found to predict reinstatement of responding following extinction of oxycodone self-administration behavior in males and females, respectively, suggesting that burst responding in M520/N females may predict high reinstatement responding during abstinence (Guha et al., 2022). Together these findings suggest that sex differences in specific self-administration metrics were strongly strain-dependent, with males in some strains showing faster session parameters and females in other strains displaying greater intake escalation or burst responding patterns, highlighting that genetic background critically shapes sex-specific drug-taking profiles that are relevant to OUD risk.

In this study, we examined patterns of oxycodone use using between-session and within-session metrics, providing detailed phenotypes for each of the tested strains. Between-session measures can reveal differences in escalation of oxycodone use, while within-session measures provide insight into dysregulated patterns of use as well as motivation to obtain oxycodone. These distinct factors of substance misuse likely have a genetic basis, and self-administration paradigms utilizing inbred animal models can unveil these genetic contributions. As genetic screening becomes more common, identifying genetic backgrounds that are susceptible to escalation and dysregulated drug use will better inform clinicians about what populations may be susceptible to OUD. Further, while all strains presented in this study escalated their oxycodone use, identification of strains that are resistant to escalation may provide additional insights into populations that can safely utilize opioids with diminished risks for abuse. Opioids are effective analgesics and may pose significantly less risks if treatments are specifically tailored to individuals with a genetic resistance to oxycodone intake escalation.

## Data Availability

The datasets presented in this article are not readily available because of ongoing studies, unpublished analyses, protection of intellectual property, or compliance with institutional and funding agency requirements. The datasets generated and analyzed during the current study may be available from the corresponding author upon reasonable request. Requests must include a clear scientific rationale and a description of the proposed analyses. Data will be provided only for non-commercial research purposes, and they may require execution of a data use agreement. Requests to access the datasets should be directed to Ryan.Bachtell@Colorado.edu.
