# Supplementary material for: Genetic modulation of oxycodone self-administration trajectories: from initiation to escalating burst patterns
Source: Front Behav Neurosci. 2026 Aug 28;20:1913370. doi: 10.3389/fnbeh.2026.1913370 (PMC13561799; doi:10.3389/fnbeh.2026.1913370)
Supplement: Supplementary file 1 [file Data_Sheet_1.pdf]

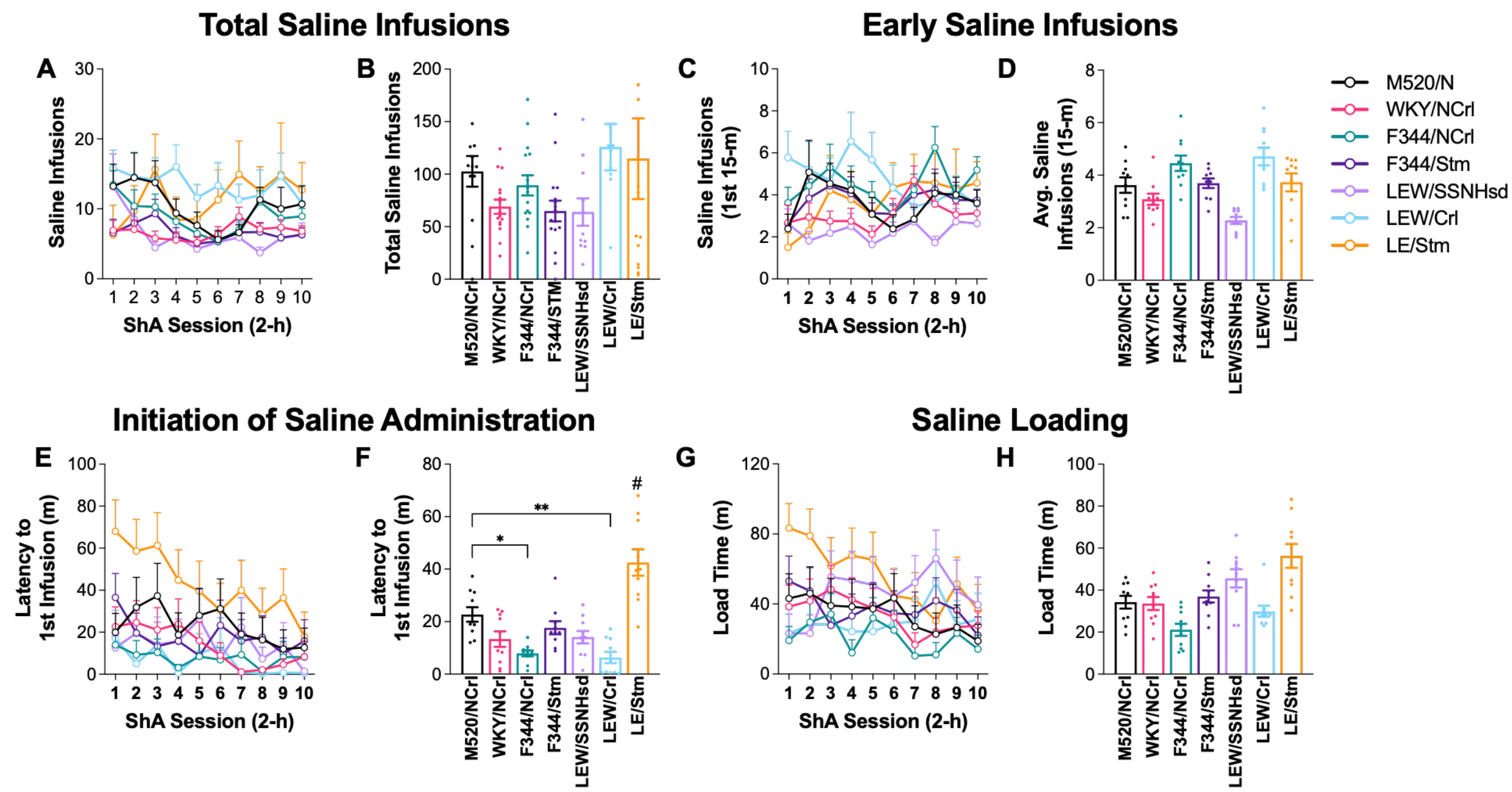

Supplemental Figure 1. Saline self-administration metrics across inbred rat strains during the acquisition phase. Mean (±SEM) saline infusions across ten 2-h ShA sessions **(A)** and total cumulative saline infusions **(B)** were comparable between the seven inbred rat strains. **(C)** Analysis of the mean (±SEM) saline intake in the first 15-m of each ShA session revealed no significant Session or Strain effects. **(D)** Overall average 15-min infusions revealed no significant strain differences. **(E)** Initiation of saline self-administration was evaluated using the latency to the 1st saline infusion. A significant main effect of Strain ($F_{6,88} = 2.99$, $p = 0.02$) and Session ($F_{2.847,250.5} = 4.57$, $p = 0.005$) indicating an overall shortening of the initiation time with the LE/Stm strain showing the longest latencies. **(F)** Overall average latency to initiate saline self-administration also highlights that the LE/Stm strain displayed significantly longer initiation times compared with all other strains (# $p < 0.05$; * $p < 0.05$; ** $p < 0.01$). **(G)** Analysis of saline loading time revealed no significant changes across LgA sessions or between strains. **(H)** Similarly, overall average loading time was not different between strains. N = 9-16/strain

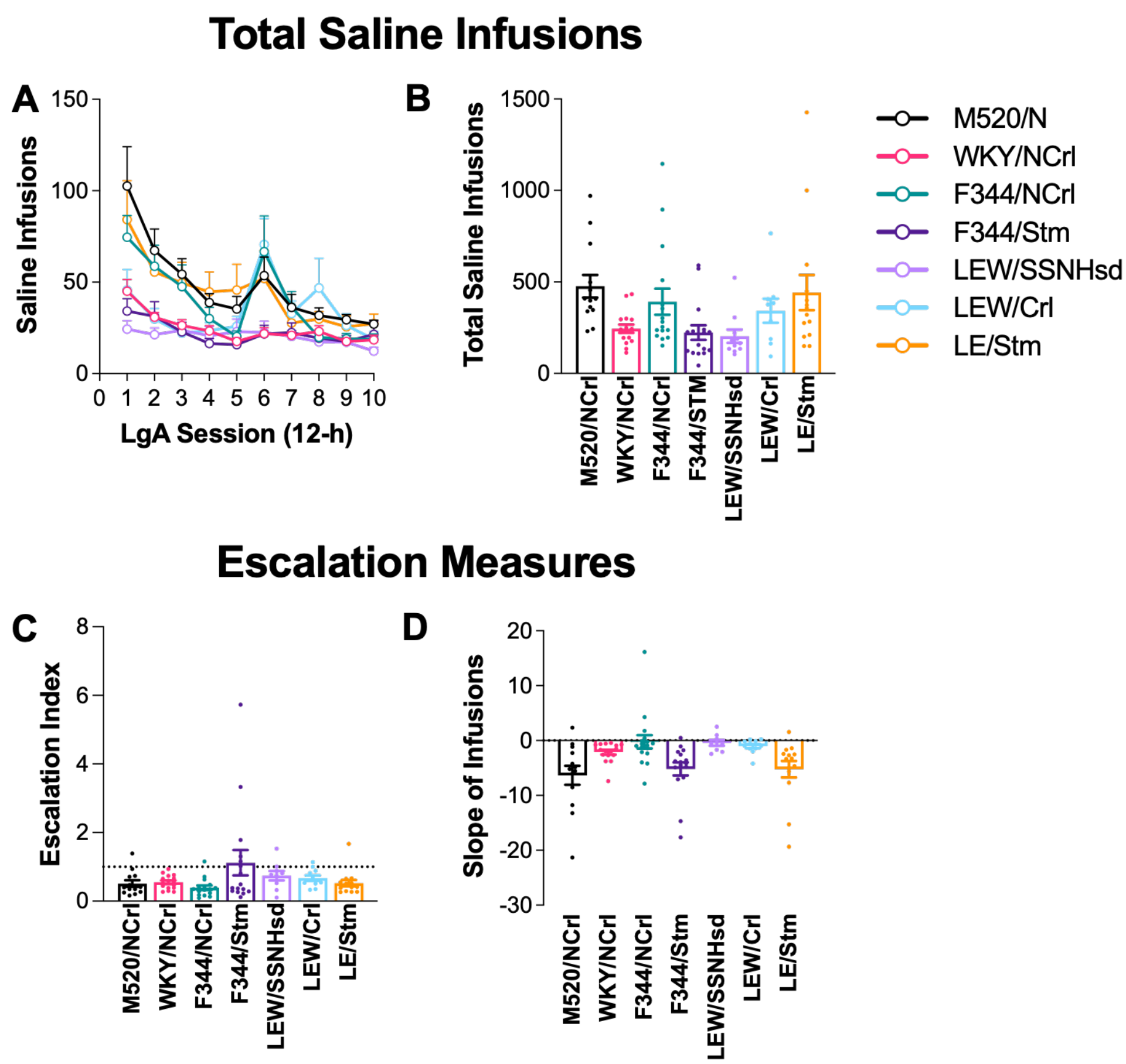

Supplemental Figure 2. Saline self-administration metrics across inbred rat strains during the escalation phase. (A) Analysis of mean (±SEM) saline infusions across ten 12-h long-access (LgA) sessions indicates a significant Sex x Session interaction where several strains displayed heightened saline intake during the first 3 LgA session followed by progressive decreased saline intake ($F_{10.78158.2}$ = 3.29, $p < 0.001$). **(B)** Total cumulative saline infusions were comparable between the seven inbred rat strains. **(C)** Analysis of the escalation index revealed no significant strain differences, with values remaining below or near 1.0 (indicating a lack of intake escalation). **(D)** Linear regression analysis of the slope of saline infusions across sessions confirmed decreasing or flat slopes across strains, demonstrating that rats did not escalate saline self-administration during extended access. N = 9-16/strain

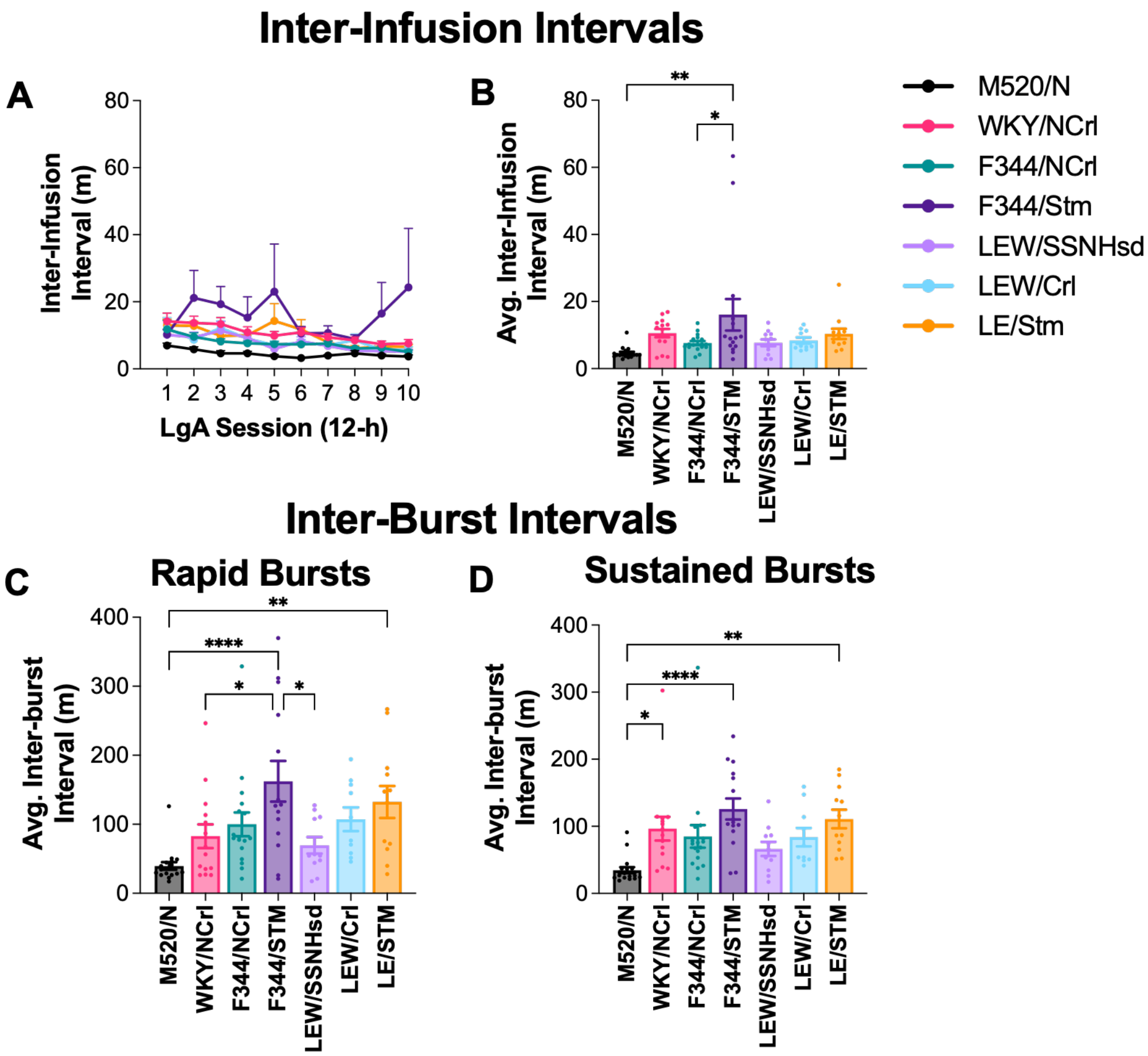

Supplemental Figure 3. Within-session inter-infusion and inter-burst intervals for oxycodone self-administration in inbred rat strains during the escalation phase. Analysis of mean (±SEM) inter-infusion interval (minutes) across ten 12-h long-access (LgA) sessions revealed progressive reductions in inter-infusion intervals across the escalation phase. **(B)** The average inter-infusion interval across the escalation phase also differed between strains with the F344/Stm strain exhibiting longer inter-infusion intervals (* $p < 0.05$; ** $p < 0.01$). Analysis of overall inter-burst intervals (IBI) for rapid **(C)** and sustained **(D)** identified significant differences between strains (Rapid IBI: $F_{6,90} = 5.43$, $p < 0.001$; Sustained IBI: $F_{6,90} = 5.14$, $p < 0.001$; * $p < 0.05$; ** $p < 0.01$; **** $p < 0.0001$). N = 10-17/strain

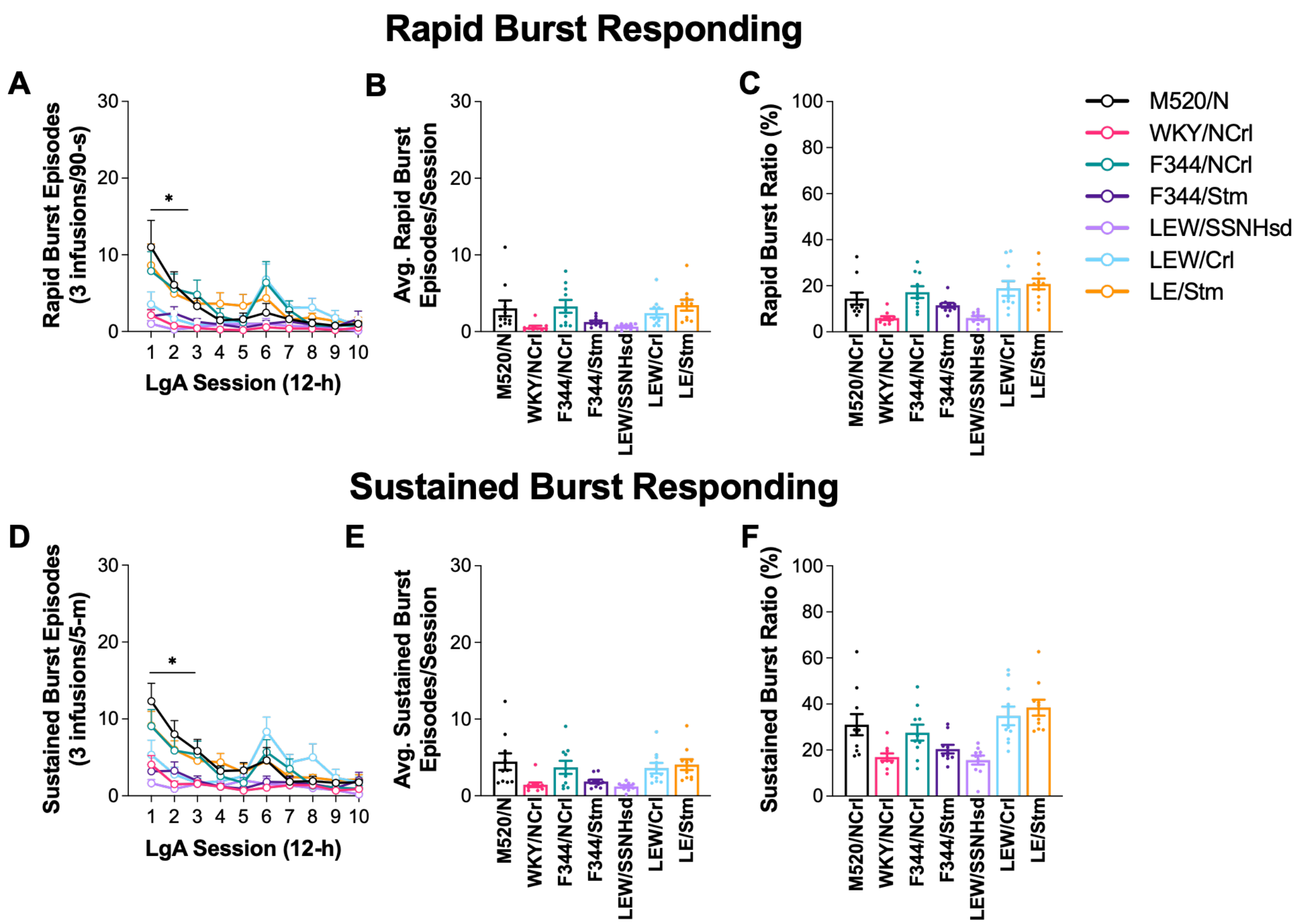

Supplemental Figure 4. Rapid and sustained burst responding for saline across inbred rat strains during the escalation phase. (A) Analysis of mean (±SEM) rapid burst episodes across ten 12-h long-access (LgA) sessions revealed a significant interaction between Strain and Session indicating that some strains displayed rapid burst responding during the first 3 LgA sessions ($F_{10.57,154.9} = 3.11$, $p = 0.001$). **(B)** The overall average rapid burst episodes per session did not differ between strains. **(C)** The rapid burst ratio (%) illustrate a low proportion of saline responding occurring within a rapid burst and was comparable in all strains. **(D)** Analysis of mean (±SEM) sustained burst episodes across ten LgA sessions revealed a significant interaction between Strain and Session indicating that some strains displayed sustained burst responding during the first 3 LgA sessions ($F_{12.05,176.8} = 3.76$, $p < 0.001$). **(E)** The overall average sustained burst episodes per session did not differ between strains. **(F)** The sustained burst ratio (%) illustrate a low proportion of saline responding occurring within a sustained bursts and was comparable in all strains. N = 9-16/strain

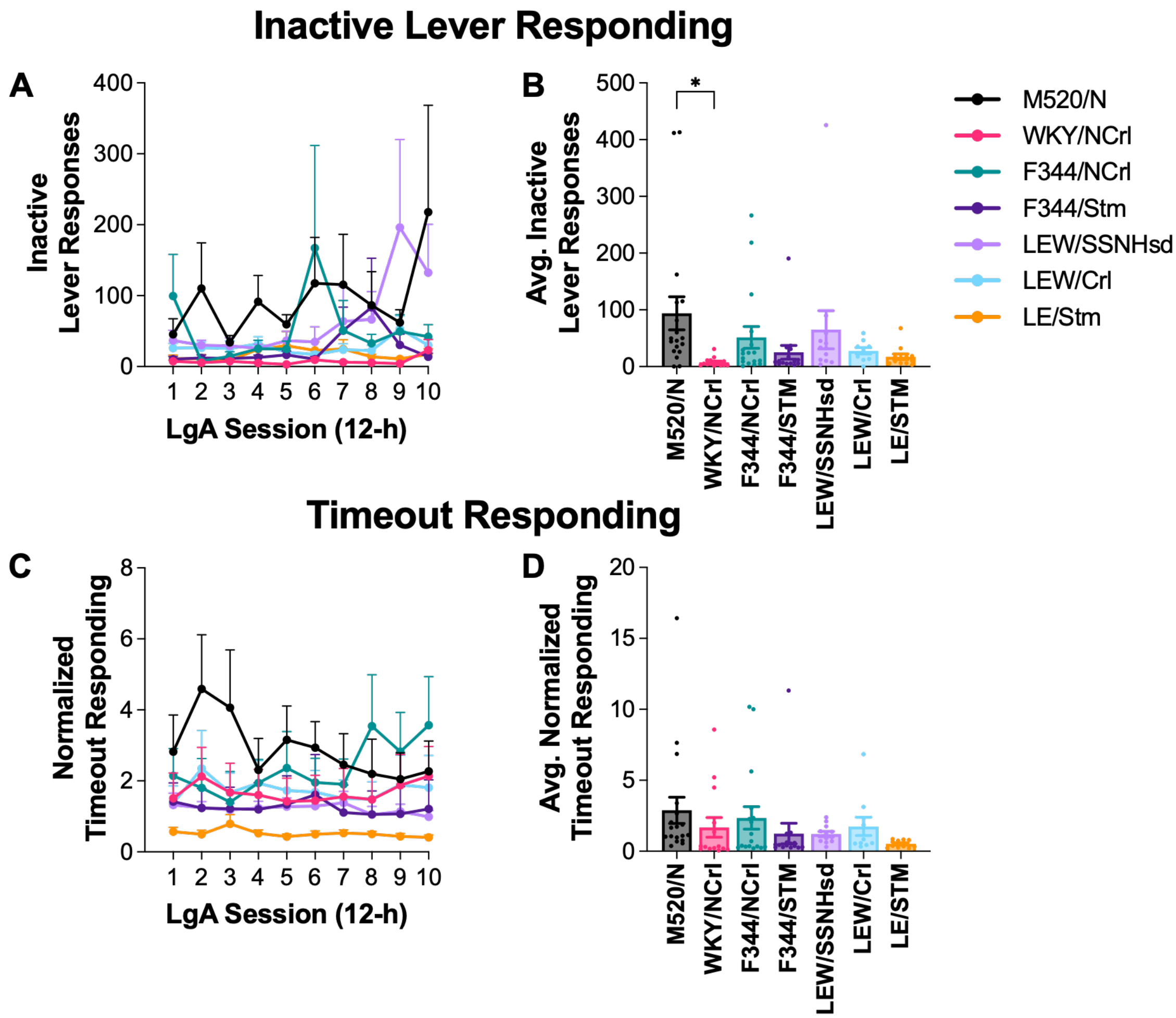

Supplemental Figure 5. Inactive lever responding and timeout responding during the escalation phase of oxycodone self-administration. (A) Mixed effects analysis of mean (±SEM) inactive lever responses across ten 12-h long-access (LgA) sessions revealed a significant main effect of Strain ($F_{6,90}$ = 2.40, p = 0.033). **(B)** Overall average inactive lever responses across the escalation phase displayed an overall effect of strain with a modest difference between the M520/N and WKY/NCrl (* $p < 0.05$). **(C)** Mixed effects analysis of the mean (±SEM) normalized timeout responding across LgA sessions revealed no significant differences between strains or sessions. **(D)** Overall average normalized timeout responding revealed no significant differences between strains across the escalation phase. N = 10-17/strain

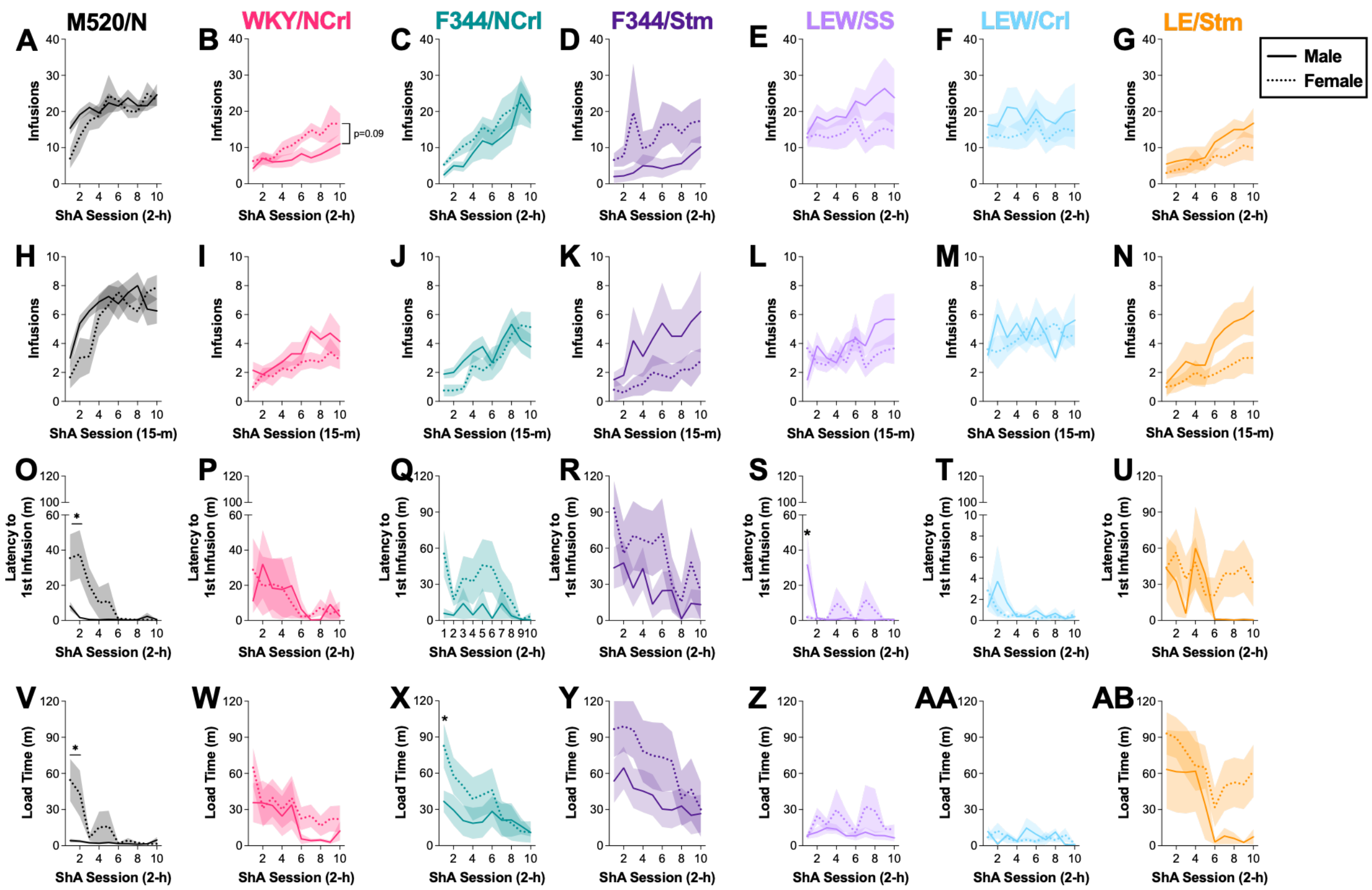

Supplemental Figure 6: Comparison between male and females in acquisition metrics. Total infusions per 2-h session during the acquisition phase is displayed for males (solid line) and females (dashed line) in the M520/N (**A**), WKY/NCrl (**B**), F344/NCrl (**C**), F344/Stm (**D**), LEW/SS (**E**), LEW/Crl (**F**), and LE/Stm (**G**) strains. No significant differences in infusions between the sexes were observed in any of the strains, although WKY/NCrl males showed a trend toward higher intake than females ($p = 0.09$). No differences between males and females were observed in the number of infusions during the first 15-m of a session is displayed for males (solid line) and females (dashed line) in all strains (panels **H-N**). The average latency to the first infusion is shown in panels **O-U**. Tukey multiple comparison test detected several subtle differences between males and females at individual Sessions in the M520/N and LEW/SS strains (* $p < 0.05$). The average load time is shown in panels **V-AB**. Tukey multiple comparison test detected several subtle differences between males and females at individual Sessions in the M520/N and F344/NCrl strains (* $p < 0.05$). All data shown as mean ± SEM.

Supplemental Table 1. Linear regression analyses for longitudinal acquisition metrics in saline self-administering rats.

Measure	Strain	Linear Regression Results
**Acquisition:** Infusions	M520/N	*b* = -0.46, *p* = 0.097
	WKY/NCrl	*b* = 0.11, *p* = 0.363
	F344/NCrl	*b* = -0.33, *p* = 0.053
	F344/Stm	*b* = -0.17, *p* = 0.283
	LEW/Ss	***b* = -0.51, *p* = 0.029***
	LEW/Crl	*b* = -0.41, *p* = 0.202
	LE/Stm	*b* = 0.61, *p* = 0.204
**Acquisition:** First 15 Minutes	M520/N	*b* = -0.02, *p* = 0.824
	WKY/NCrl	*b* = 0.10, *p* = 0.157
	F344/NCrl	*b* = 0.08, *p* = 0.339
	F344/Stm	*b* = 0.05 *p* = 0.553
	LEW/Ss	*b* = 0.03, *p* = 0.597
	LEW/Crl	***b* = -0.26, *p* = 0.027***
	LE/Stm	***b* = 0.29, *p* = 0.040***
**Acquisition:** Latency to 1st infusion	M520/N	*b* = -1.85, *p* = 0.147
	WKY/NCrl	***b* = -2.67, *p* = 0.002***
	F344/NCrl	*b* = -0.53, *p* = 0.439
	F344/Stm	*b* = -1.32, *p* = 0.208
	LEW/Ss	*b* = -1.05, *p* = 0.318
	LEW/Crl	*b* = -1.48, *p* = 0.087
	LE/Stm	***b* = -4.82, *p* = 0.002***
**Acquisition:** Load Time	M520/N	*b* = -2.82, *p* = 0.055
	WKY/NCrl	***b* = -2.46, *p* = 0.037***
	F344/NCrl	*b* = -1.31, *p* = 0.213
	F344/Stm	*b* = -1.74, *p* = 0.204
	LEW/Ss	*b* = 2.19, *p* = 0.185
	LEW/Crl	*b* = 1.29, *p* = 0.437
	LE/Stm	***b* = -5.24, *p* = 0.002***

Significance indicated by emboldened text and asterisk (*, $p < 0.05$).

Supplemental Table 2. Linear regression analyses for longitudinal escalation metrics in saline self-administering rats.

Measure	Strain	Linear Regression Results
**Escalation:** Infusions	M520/N	***b* = -6.34, *p* < 0.001***
	WKY/NCrl	***b* = -2.12, *p* < 0.001***
	F344/NCrl	***b* = -5.17, *p* < 0.001***
	F344/Stm	***b* = -1.24, *p* = 0.035***
	LEW/Ss	***b* = -1.03, *p* = 0.033***
	LEW/Crl	*b* = -0.43, *p* = 0.695
	LE/Stm	***b* = -5.24, *p* < 0.001***
**Escalation:** Inter-infusion intervals	M520/N	***b* = 1.96, *p* < 0.001***
	WKY/NCrl	***b* = 2.65, *p* < 0.001***
	F344/NCrl	***b* = 4.25, *p* < 0.001***
	F344/Stm	***b* = 4.12, *p* = 0.006***
	LEW/Ss	***b* = 2.99, *p* = 0.003***
	LEW/Crl	*b* = 1.48, *p* = 0.295
	LE/Stm	***b* = 2.46, *p* < 0.001***
**Escalation:** Rapid Burst Episodes	M520/N	***b* = -0.83, *p* < 0.001***
	WKY/NCrl	***b* = -0.11, *p* < 0.001***
	F344/NCrl	***b* = -0.67, *p* < 0.001***
	F344/Stm	*b* = -0.10, *p* = 0.149
	LEW/Ss	*b* = -0.04, *p* = 0.432
	LEW/Crl	*b* = 0.06, *p* = 0.692
	LE/Stm	***b* = -0.64, *p* < 0.001***
**Escalation:** Sustained Burst Episodes	M520/N	***b* = -0.98, *p* < 0.001***
	WKY/NCrl	***b* = -0.21, *p* < 0.001***
	F344/NCrl	***b* = -0.73, *p* < 0.001***
	F344/Stm	*b* = -0.16, *p* = 0.053
	LEW/Ss	*b* = -0.10, *p* = 0.140
	LEW/Crl	*b* = -0.03, *p* = 0.832
	LE/Stm	***b* = -0.65, *p* < 0.001***

Significance indicated by emboldened text and asterisk (*, p < 0.05).
